# Supplementary material for: Precise synthesis and photovoltaic properties of giant molecule acceptors
Source: Nat Commun. 2023 Dec 2;14:7996. doi: 10.1038/s41467-023-43846-3 (PMC10693637; doi:10.1038/s41467-023-43846-3)
Supplement: Supplementary file 1 — Supplementary Information [file 41467_2023_43846_MOESM1_ESM.pdf]

Supplementary Information

Precise synthesis and photovoltaic properties of giant molecule acceptors

Hongmei Zhuo,[1,2,6] Xiaojun Li,[1,2,6]* Jinyuan Zhang,[1,6] Can Zhu,[1,2] Haozhe He,[1,2] Kan Ding,[3] Jing Li,[4] Lei Meng,[1,2] Harald Ade,[3]* Yongfang Li[1,2,5]*

[1] *Beijing National Laboratory for Molecular Sciences, CAS Key Laboratory of Organic Solids, Institute of Chemistry, Chinese Academy of Sciences, Beijing 100190, China.*

[2] *School of Chemical Science, University of Chinese Academy of Sciences, Beijing 100049, China.*

[3] *Department of Physics and Organic and Carbon Electronics Lab (ORaCEL), North Carolina State University, Raleigh, North Carolina 27695, USA.*

[4] *Key Laboratory of Photochemical Conversion and Optoelectronic Materials, Technical Institute of Physics and Chemistry, Chinese Academy of Sciences, Beijing 100190, China.*

[5] *Laboratory of Advanced Optoelectronic Materials, College of Chemistry, Chemical Engineering and Materials Science, Soochow University, Suzhou, Jiangsu 215123, China.*

[6] These authors contributed equally: Hongmei Zhuo, Xiaojun Li, Jinyuan Zhang

Supplementary Methods

Synthesis of YDT, m1 and m2

BTCHO (1 g, 0.678 mmol) and the terminal groups IC (145 mg, 0.746 mmol) and IC-Br (203 mg, 0.746 mmol) were dissolved in toluene (50 mL). 5 mL Boron trifluoride diethyl etherate, 48% BF_3 and 2 mL anhydrous acetic anhydride were also added separately in this system. After stirring for 6 h, the mixture was poured into methanol and filtered. The residue was purified with column chromatography on silica gel using dichloromethane/petroleum ether (1/3) as the eluent. Finally, yielding the dark blue solids of YDT (338 mg, 27.3% yield), m1 (274 mg, 20.4% yield) and m2 (605 mg, 46.9% yield). YDT: ^{1}H NMR (400 MHz, $CDCl_3$) δ 9.17 (s, 2H), 8.70 (d, *J* = 6.7 Hz, 2H), 7.96 (d, *J* = 6.2 Hz, 2H), 7.80-7.73 (m, 4H), 4.78 (d, *J* = 7.8 Hz, 4H), 3.23 (t, *J* = 7.9 Hz, 4H), 2.16 (t, *J* = 6.5 Hz, 2H), 1.88 (t, *J* = 7.8 Hz, 4H), 1.52 (d, *J* = 7.8 Hz, 4H), 1.31-0.79 (m, 125H). ^{13}C NMR (101 MHz, $CDCl_3$) δ 188.45, 160.87, 153.04, 147.46, 144.91, 140.07, 137.77, 136.93, 135.44, 135.01, 134.20, 133.91, 133.29, 130.16, 125.26, 123.56, 120.82, 115.37, 114.96, 113.46, 77.35, 77.03, 76.71, 68.24, 55.64, 39.12, 31.95, 31.22, 30.51, 29.81, 29.70, 29.58, 29.46, 25.55, 22.71, 14.18, 14.09. HRMS (MALDI-TOF) m/z calcd for $[M]^+$ $C_{114}H_{154}N_8O_2S_5$ 1827.07, found 1827.07.

m2: ^{1}H NMR (400 MHz, $CDCl_3$) δ 9.17 (s, 2H), 8.71 (d, *J* = 6.9 Hz, 1H), 8.54 (d, *J* = 8.4 Hz, 1H), 8.03 (s, 1H), 7.98-7.93 (m, 1H), 7.84 (d, *J* = 8.4 Hz, 1H), 7.79-7.74 (m, 2H), 4.78 (d, *J* = 7.8 Hz, 4H), 3.22 (d, *J* = 8.0 Hz, 4H), 2.15 (t, *J* = 6.5 Hz, 2H), 1.93-1.82 (m, 4H), 1.56-1.47 (m, 4H), 1.32-0.83 (m, 125H). ^{13}C NMR (101 MHz, $CDCl_3$) δ 188.06, 186.87, 147.50, 140.11, 138.55, 138.37, 137.60, 136.97, 135.02, 134.21, 130.87, 130.20, 129.42, 126.71, 126.38, 125.24, 123.57, 115.30, 114.92, 77.34, 77.02, 76.71, 55.77, 55.69, 39.16, 31.97, 31.94, 31.22, 30.61, 29.91, 29.81, 29.78, 29.71, 29.69, 29.65, 29.63, 29.57, 29.50, 29.48, 29.44, 29.41, 29.37, 29.34, 25.65, 25.60, 22.70, 14.11. HRMS (MALDI-TOF) m/z calcd for $[M]^+$ $C_{114}H_{153}BrN_8O_2S_5$ 1904.99, found 1904.99.

Synthesis of PY-IT

2,5-bis(trimethylstannyl)thiophene monomer (12.3 mg, 0.03 mmol) and bromide BT2ICBr (60 mg, 0.03 mmol) were dissolved in toluene (3 mL). $Pd_2(dba)_3$ (1.7 mg,

0.0019 mmol) and P(o-tolyl)$_3$ (1.3 mg, 0.0044 mmol) were also added into the system after being flushed with argon for five minutes. Then, the reaction mixtures were purged with argon for another 15 min. The reactions were stirred at 110 °C for 24 h. The polymers were precipitated in methanol (100 mL) and filtrated. The dried precipitates were purified by Soxhlet extractor and then subjected to Soxhlet extractions with methanol, hexane, acetone, and chloroform, respectively. Then the chloroform fraction was concentrated and precipitated with methanol, the black solids PY-IT was obtained. (36 mg, 60% yield). M_n = 8210, PDI = 2.08.

Synthesis of m3

BTCHO (500 mg, 0.339 mmol) and IC-Br (101.4 mg, 0.373 mmol) were dissolved in toluene (60 mL). 3 mL Boron trifluoride diethyl etherate, 48% BF_3 and 1 mL anhydrous acetic anhydride were also added separately in this system. After stirring for 5 h, the mixture was poured into methanol and filtered. The residue was purified with column chromatography on silica gel using dichloromethane/petroleum ether (1/3) as the eluent. Finally, yielding the green solid of m3 (352.8 mg, 60.2% yield). ^{1}H NMR (400 MHz, $CDCl_3$) δ 10.16 (s, 1H), 9.17 (s, 1H), 8.56 (d, J = 8.4 Hz, 1H), 8.02 (d, J = 1.5 Hz, 1H), 7.84 (dd, J = 8.3, 1.9 Hz, 1H), 4.69 (dd, J = 32.0, 7.7 Hz, 4H), 3.21 (dd, J = 7.5, 3.0 Hz, 4H), 2.08 (d, J = 6.1 Hz, 2H), 1.91 (dt, J = 23.2, 7.6 Hz, 4H), 1.55-1.45 (m, 4H), 1.31-0.84 (m, 125H). ^{13}C NMR (101 MHz, $CDCl_3$) δ 187.10, 181.83, 160.18, 153.95, 147.75, 147.58, 146.82, 144.93, 143.91, 138.70, 138.52, 137.70, 137.63, 137.32, 137.19, 136.46, 135.78, 134.98, 133.36, 132.54, 131.21, 129.58, 129.50, 127.58, 126.81, 126.53, 119.93, 115.48, 115.12, 113.63, 112.82, 77.48, 77.16, 76.84, 68.20, 55.82, 55.53, 39.30, 39.06, 32.10, 32.06, 31.40, 30.82, 30.54, 30.48, 30.04, 30.00, 29.87, 29.82, 29.79, 29.76, 29.73, 29.70, 29.67, 29.61, 29.57, 29.53, 29.50, 29.49, 29.46, 29.44, 28.35, 25.85, 25.52, 22.85, 22.83, 14.26, 14.25. HRMS (MALDI-TOF) m/z calcd for $[M]^+$ $C_{102}H_{149}BrN_6O_2S_5$ 1728.95, found 1728.95.

Synthesis of m4

m2 (350 mg, 0.184 mmol), m3 (318.1 mg, 0.184 mmol) and 2,5-bis(trimethylstannyl)thiophene (68.4 mg, 0.167 mmol) were added in 30 mL toluene.

Then, $Pd_2(dba)_3$ (5 mg, 0.0055 mmol) and $P(o\text{-tolyl})_3$ (3.9 mg, 0.0127 mmol) were also added into the system after being flushed with argon for five minutes. Then, the reaction mixtures were purged with argon for another 15 min. The reactions were stirred at 110 °C for 10 h. The mixture was poured into methanol and filtered. The residue was purified with column chromatography on silica gel using dichloromethane/petroleum ether (1/2) as the eluent. Finally, yielding the dark green solid of m4 (239.5 mg, 40.3% yield). ^{1}H NMR (700 MHz, $CDCl_3$) δ 10.16 (s, 1H), 9.16 (s, 3H), 8.73 (dd, *J* = 24.5, 7.7 Hz, 3H), 8.15 (s, 1H), 8.12-8.03 (m, 1H), 7.97 (dd, *J* = 11.3, 7.5 Hz, 3H), 7.77 (dd, *J* = 10.9, 7.1 Hz, 2H), 7.64 (s, 2H), 4.84-4.74 (m, 6H), 4.68 (d, *J* = 7.7 Hz, 2H), 3.21 (t, *J* = 7.8 Hz, 8H), 2.23-2.09 (m, 4H), 1.97-1.85 (m, 8H), 1.52 (s, 10H), 1.28-0.81 (m, 250H). ^{13}C NMR (176 MHz, $CDCl_3$) δ 188.40, 187.97, 181.72, 160.83, 159.90, 153.55, 153.35, 153.00, 147.60, 147.50, 147.44, 146.75, 145.01, 144.79, 143.93, 143.72, 140.06, 139.04, 137.90, 137.42, 136.94, 136.05, 135.62, 135.39, 135.16, 135.04, 134.62, 134.23, 134.02, 133.84, 133.29, 132.45, 131.40, 130.76, 130.50, 130.14, 129.46, 128.98, 128.41, 127.40, 127.21, 126.09, 125.44, 125.23, 123.58, 120.86, 120.50, 119.08, 115.50, 115.38, 115.21, 114.92, 113.53, 113.39, 112.78, 77.20, 77.17, 77.02, 76.99, 76.84, 76.81, 68.29, 67.61, 67.32, 55.69, 55.38, 39.14, 39.10, 39.05, 38.89, 31.96, 31.24, 30.56, 30.41, 30.33, 30.28, 29.92, 29.84, 29.72, 29.67, 29.60, 29.56, 29.51, 29.41, 29.37, 28.21, 25.65, 25.38, 22.71, 14.14. HRMS (MALDI-TOF) m/z calcd for $[M]^+$ $C_{220}H_{304}N_{14}O_4S_{11}$ 3558.09, found 3558.09.

Synthesis of m5

m4 (220 mg, 0.062 mmol) and the terminal group IC-Br (20.2 mg, 0.074 mmol) were dissolved in toluene (20 mL), 0.2 mL Boron trifluoride diethyl etherate, 48% BF_3 and 0.1 mL anhydrous acetic anhydride were also added separately in this system. After stirring for 5 h, the mixture was poured into methanol and filtered. The residue was purified with column chromatography on silica gel using dichloromethane/petroleum ether (1/2) as the eluent. Finally, yielding the dark blue solid of m5 (218.2 mg, 92.3% yield). ^{1}H NMR (400 MHz, $CDCl_3$) δ 9.15-9.03 (m, 4H), 8.71 (t, *J* = 6.6 Hz, 3H), 8.54 (d, *J* = 8.4 Hz, 1H), 8.07 (s, 2H), 7.95 (d, *J* = 12.1 Hz, 3H), 7.84 (dd, *J* = 13.3, 7.9 Hz,

2H), 7.76 (t, J = 7.7 Hz, 1H), 7.65 (s, 3H), 4.83 (d, J = 7.9 Hz, 8H), 3.19 (dt, J = 28.3, 9.0 Hz, 8H), 2.31-2.16 (m, 4H), 1.87 (dd, J = 15.0, 7.5 Hz, 8H), 1.54 (d, J = 7.0 Hz, 10H), 1.31-0.83 (m, 250H). ^{13}C NMR (101 MHz, $CDCl_3$) δ 188.23, 187.75, 186.74, 159.68, 153.53, 153.33, 152.94, 147.53, 145.14, 143.95, 143.89, 140.03, 139.02, 138.46, 138.26, 137.91, 137.74, 137.55, 136.89, 136.02, 134.96, 134.25, 134.07, 133.50, 131.18, 130.78, 130.56, 130.22, 129.42, 127.22, 126.36, 126.05, 125.23, 123.47, 120.82, 120.01, 119.08, 115.46, 115.15, 114.86, 113.78, 113.63, 113.53, 77.34, 77.03, 76.71, 68.41, 68.33, 67.68, 55.83, 39.22, 31.96, 31.24, 30.63, 29.95, 29.90, 29.83, 29.73, 29.68, 29.66, 29.62, 29.57, 29.53, 29.49, 29.42, 29.38, 25.71, 22.71, 14.12, 0.00. HRMS (MALDI-TOF) m/z calcd for $[M]^+$ $C_{232}H_{307}BrN_{16}O_4S_{11}$ 3812.04, found 3812.04.

Synthesis of DY, TY and QY

m5 (210 mg, 0.055 mmol), m2 (104.8 mg, 0.055 mmol) and 2,5-bis(trimethylstannyl)thiophene (20.5 mg, 0.05 mmol) were added in 30 mL toluene. Then, $Pd_2(dba)_3$ (2.3 mg, 0.0025 mmol) and P(o-tolyl)$_3$ (1.8 mg, 0.0058 mmol) were also added into the system after being flushed with argon for five minutes. Then, the reaction mixtures were purged with argon for another 15 min. The reactions were stirred at 110 °C for 10 h. The mixture was poured into methanol and filtered. The residue was purified with column chromatography on silica gel using dichloromethane/petroleum ether (1/1) as the eluent. Finally, yielding the dark solids of DY (44.1 mg, 23.6% yield), TY (60.4 mg, 21.4% yield) and QY (68.3 mg, 18.1% yield). DY: ^{1}H NMR (700 MHz, $CDCl_3$) δ 9.16 (d, J = 11.6 Hz, 4H), 8.75 (dd, J = 15.9, 7.7 Hz, 4H), 8.19-8.08 (m, 2H), 8.00 (d, J = 8.1 Hz, 2H), 7.93 (d, J = 7.1 Hz, 2H), 7.79 (t, J = 7.4 Hz, 2H), 7.72 (t, J = 7.2 Hz, 2H), 7.68 (s, 2H), 4.83 (d, J = 7.9 Hz, 8H), 3.23 (d, J = 23.8 Hz, 8H), 2.27-2.16 (m, 4H), 1.91 (dd, J = 14.6, 7.3 Hz, 8H), 1.59-1.50 (m, 10H), 1.43-0.80 (m, 250H). ^{13}C NMR (176 MHz, $CDCl_3$) δ 188.32, 187.82, 160.75, 159.78, 153.38, 152.97, 147.53, 147.50, 145.09, 144.98, 143.93, 140.05, 139.01, 137.91, 137.86, 137.82, 136.91, 135.68, 135.39, 134.98, 134.11, 133.86, 133.51, 133.33, 130.56, 130.15, 127.21, 126.07, 125.22, 123.49, 120.86, 120.73, 119.13, 115.46, 115.35, 115.15, 114.88, 113.57, 113.53, 77.19, 77.01, 76.82, 68.36, 67.56, 55.72, 39.14, 31.94, 31.92, 31.24,

31.18, 30.54, 29.92, 29.89, 29.85, 29.82, 29.78, 29.72, 29.70, 29.68, 29.66, 29.64, 29.59, 29.56, 29.51, 29.49, 29.46, 29.40, 29.37, 29.36, 29.34, 25.62, 22.70, 22.69, 22.68, 14.11, 14.10. HRMS (MALDI-TOF) m/z calcd for $[M]^+$ $C_{232}H_{308}N_{16}O_4S_{11}$ 3734.13, found 3734.13.

TY: ^{1}H NMR (400 MHz, $CDCl_3$) δ 9.35-8.49 (m, 11H), 8.21-7.30 (m, 17H), 4.92 (s, 12H), 3.06 (s, 12H), 2.28 (s, 6H), 1.75-0.71 (m, 415H). ^{13}C NMR (101 MHz, $CDCl_3$) δ 187.58, 153.39, 147.62, 145.10, 143.72, 140.04, 139.00, 137.92, 137.63, 135.77, 134.79, 134.15, 133.59, 133.35, 130.62, 127.07, 125.94, 125.27, 120.51, 115.54, 115.26, 115.00, 113.56, 113.41, 77.35, 77.03, 76.71, 68.30, 67.51, 56.03, 39.23, 31.96, 31.10, 30.75, 29.92, 29.78, 29.74, 29.69, 29.61, 29.60, 29.54, 29.50, 29.42, 29.38, 25.85, 22.71, 22.69, 14.13, 14.10. HRMS (MALDI-TOF) m/z calcd for $[M]^+$ $C_{350}H_{463}N_{24}O_6S_{17}$ 5642.19, found 5642.19.

QY: ^{1}H NMR (400 MHz, $CDCl_3$) δ 9.23 (s, 4H), 9.04-8.68 (m, 13H), 8.28 (s, 2H), 8.10-7.85 (m, 8H), 7.79 (d, *J* = 10.7 Hz, 7H), 7.44 (d, *J* = 32.4 Hz, 9H), 5.09-4.77 (m, 16H), 3.26 (s, 11H), 2.90-2.46 (m, 8H), 2.44-2.16 (m, 10H), 1.92 (s, 11H), 1.33-0.82 (m, 559H). ^{13}C NMR (101 MHz, $CDCl_3$) δ 187.38, 159.57, 153.84, 147.66, 145.01, 143.79, 140.12, 139.08, 138.15, 137.85, 137.01, 136.32, 135.68, 134.26, 133.45, 130.65, 126.59, 120.93, 120.12, 115.53, 113.58, 113.41, 77.33, 77.01, 76.69, 68.31, 67.66, 55.81, 39.17, 31.97, 31.24, 30.75, 29.72, 29.42, 25.83, 22.72, 14.13, 1.02. HRMS (MALDI-TOF) m/z calcd for $[M]^+$ $C_{468}H_{618}N_{32}O_8S_{23}$ 7550.25, found 7550.25.

Supplementary Figures

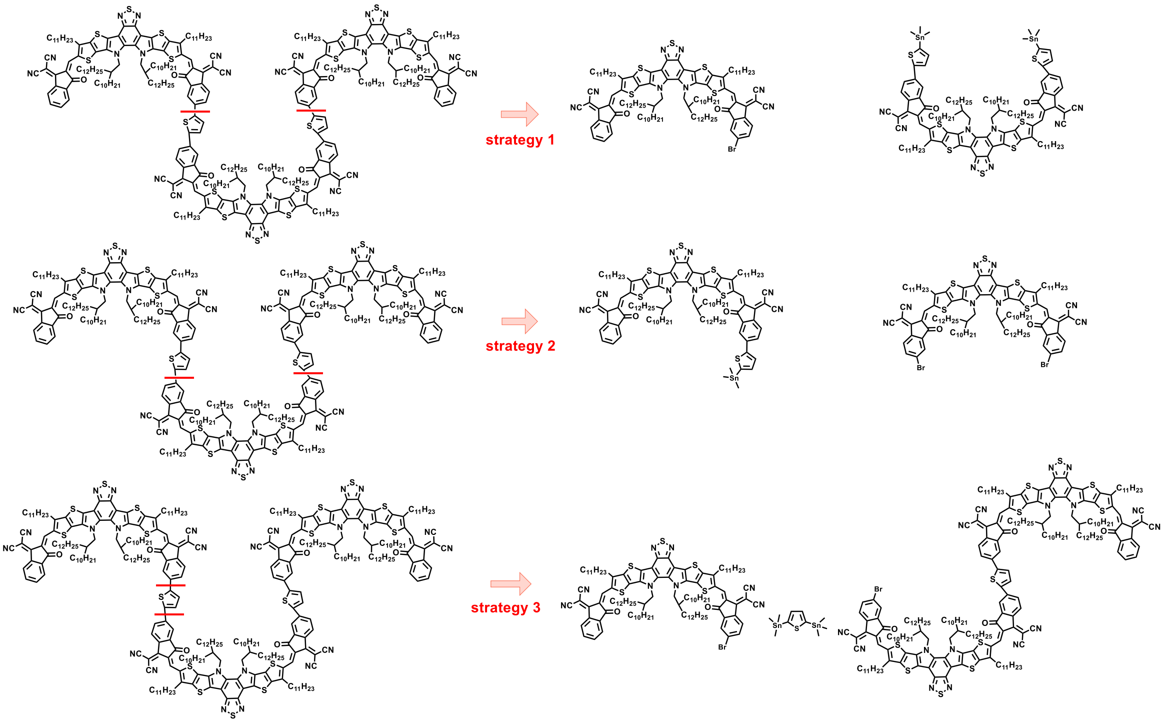

Supplementary Fig. 1. The retrosynthetic analysis of TY.

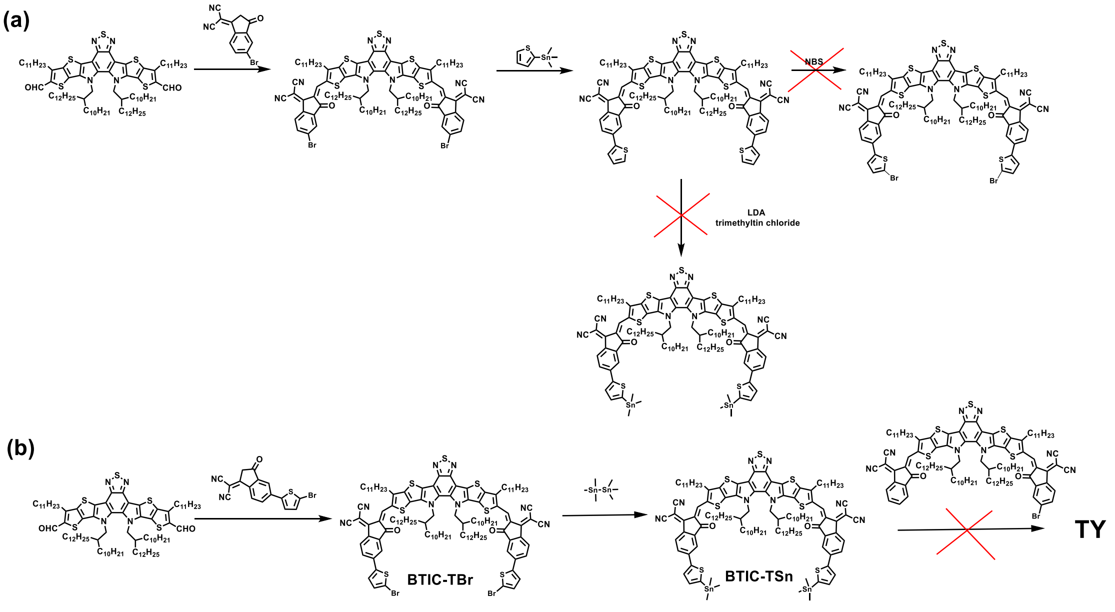

Supplementary Fig. 2. The failed synthetic methods of TY. (a) The failed synthetic route though adding tin or bromo groups to the thiophene bridge. (b) The failed synthetic route though synthesizing the end groups with bromo substituted thiophene bridge.

It seems that after connecting the thiophene linking unit to the SMA part, further modification of the α position of the thiophene unit becomes very difficult (Supplementary Fig. 2a). In addition, we also tried to directly synthesize the terminal group with the bromine-modified thiophene. After the coupling of hexamethyldistannane and bromine-terminated BTIC-TBr, the compound BTIC-TSn was successfully prepared. But unfortunately, we did not obtain the GMA TY through this process (Supplementary Fig. 2b)

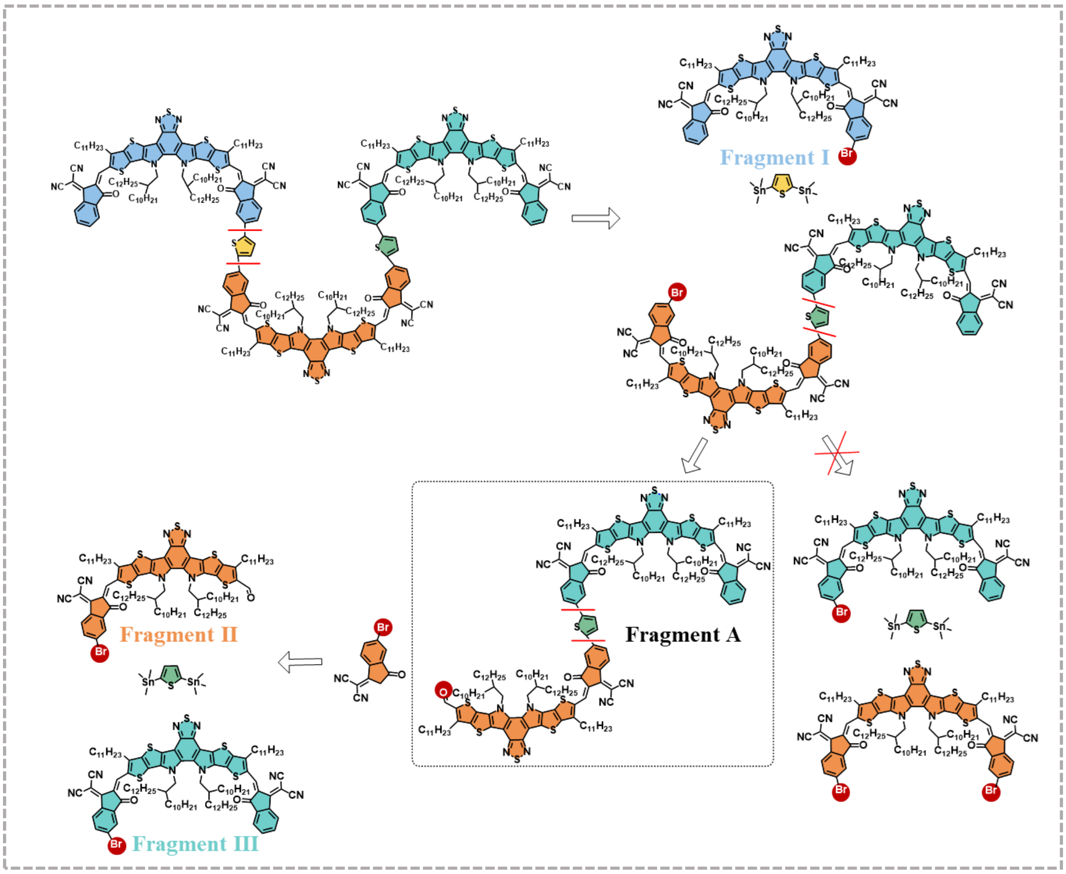

Supplementary Fig. 3. The detailed preparation of TY by asymmetric disconnection strategy. (Fragments I, II and III represent the first, second and third synthetic fragment to get TY. Fragment A refers to the middle fragment obtained from Fragments II and III coupling with thiophene bridge)

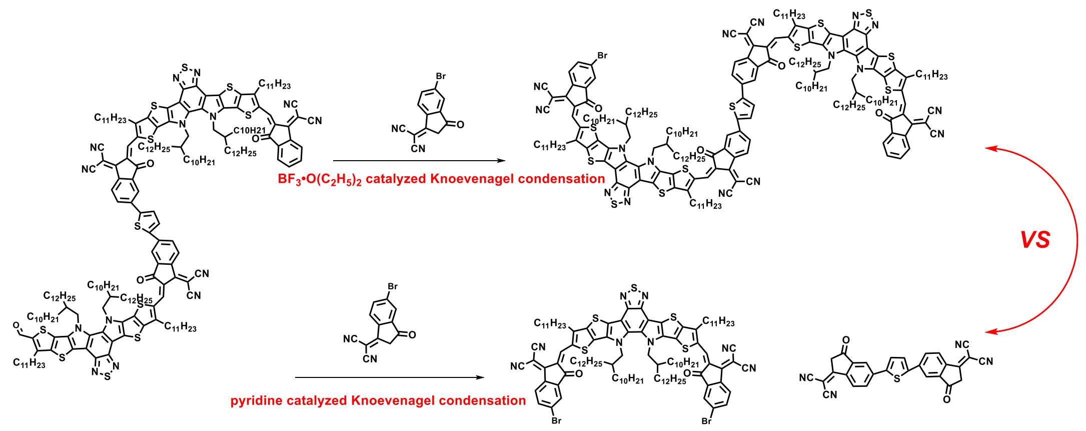

Supplementary Fig. 4. The comparation between boron trifluoride etherate and pyridine catalyzed Knoevenagel condensation.

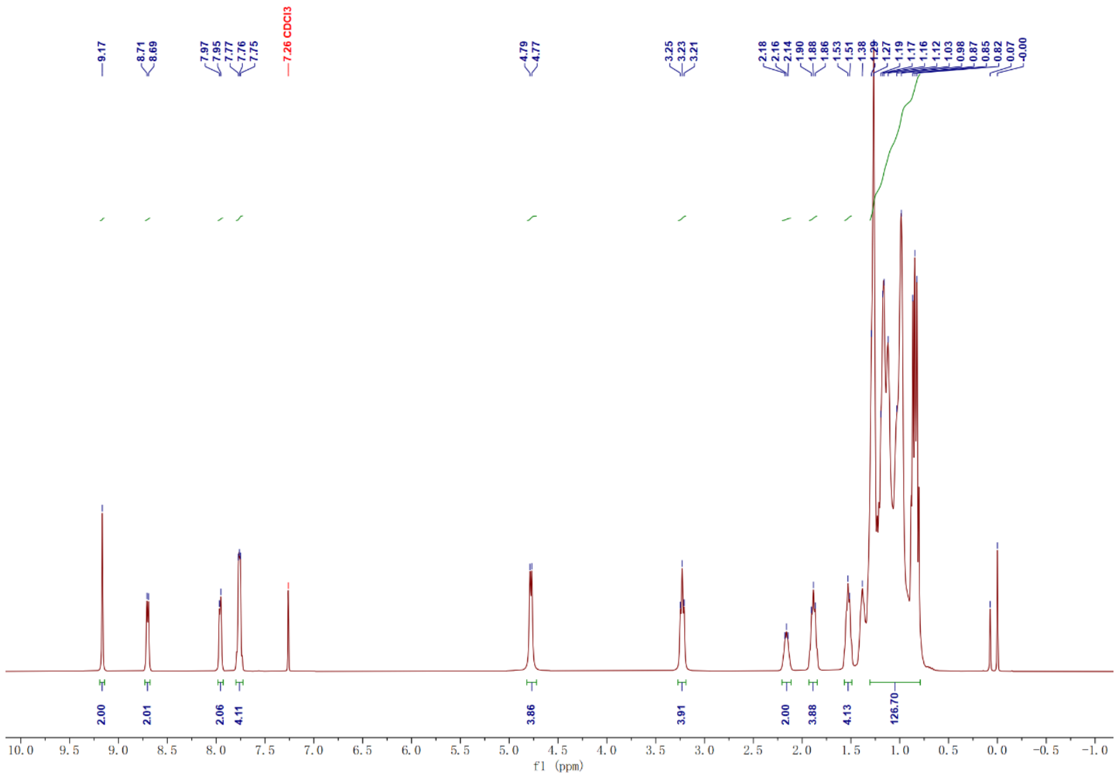

Supplementary Fig. 5. ^{1}H NMR spectrum of YDT in $CDCl_3$.

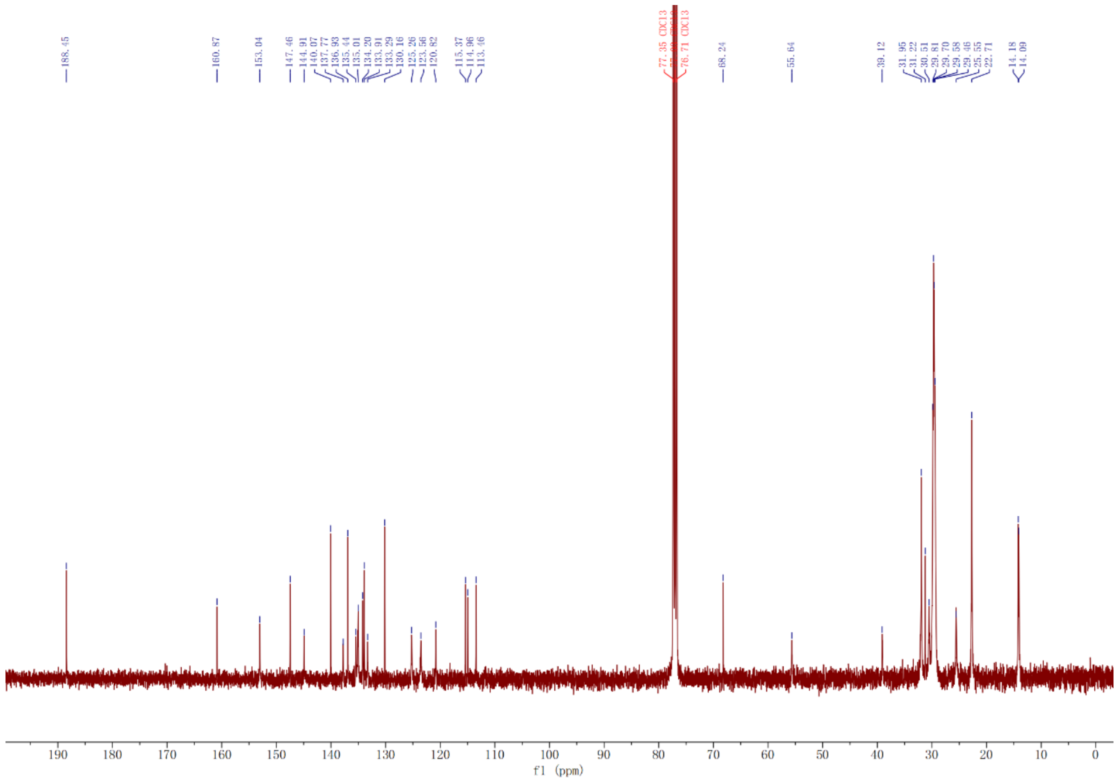

Supplementary Fig. 6. ^{13}C NMR spectrum of YDT in $CDCl_3$.

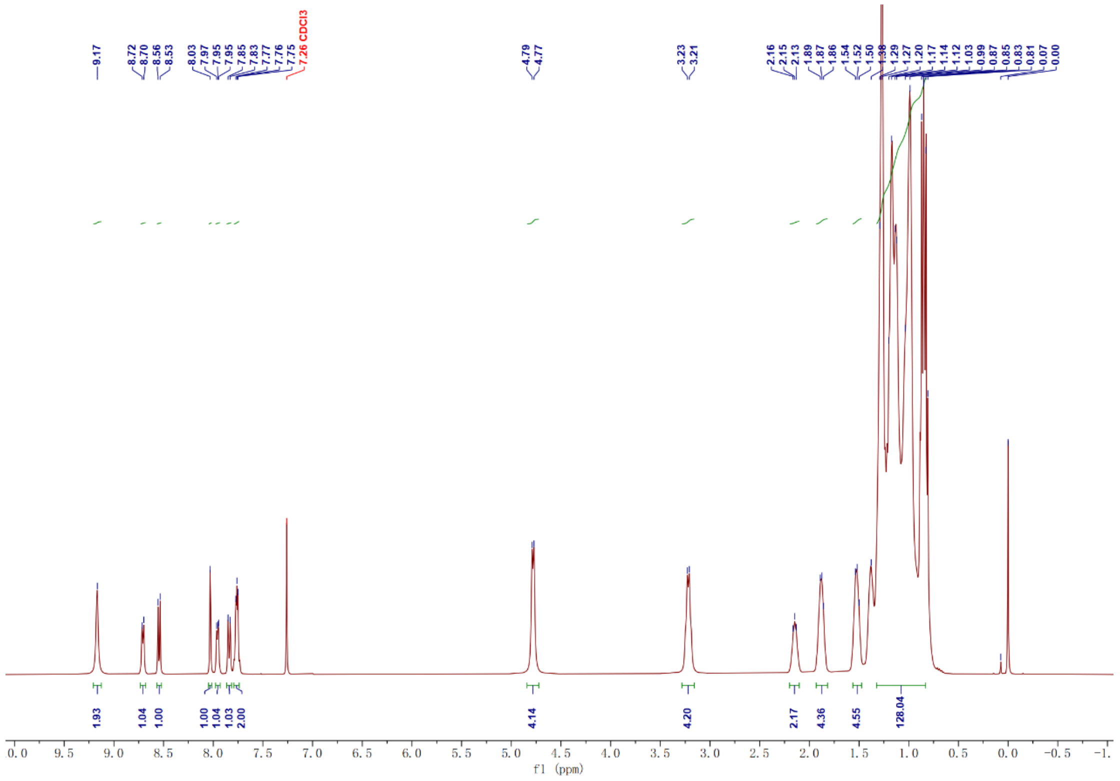

Supplementary Fig. 7. ^{1}H NMR spectrum of BTIC-ICBr in $CDCl_3$.

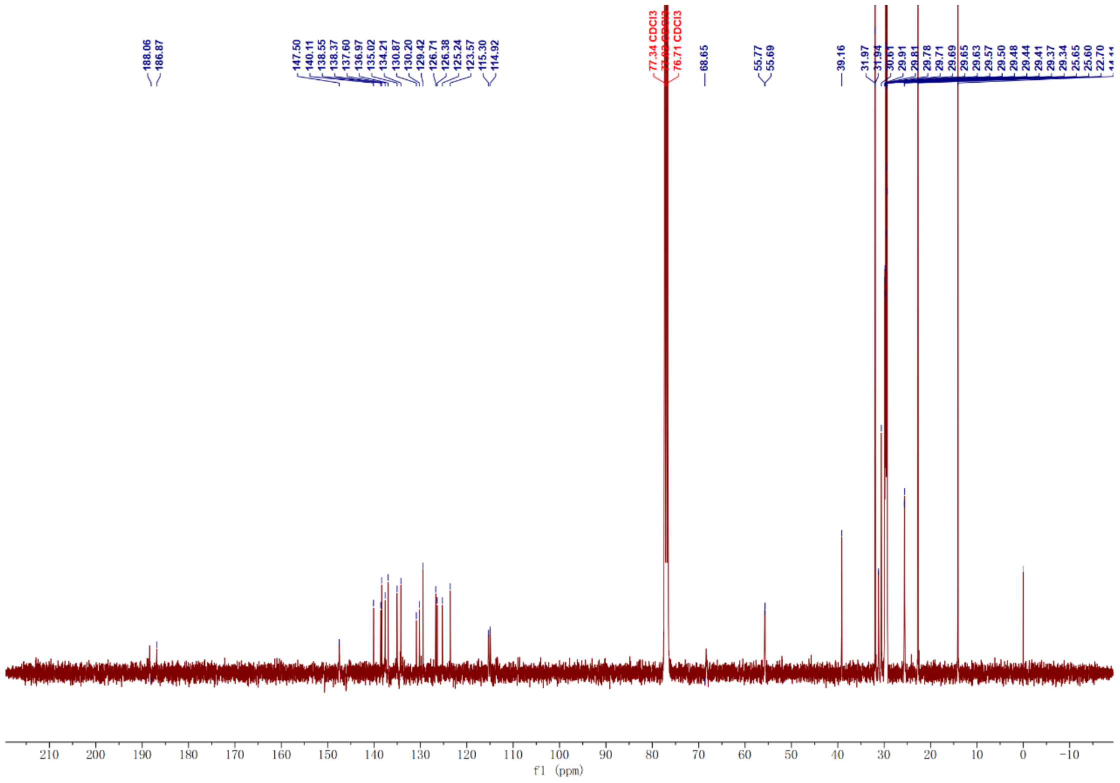

Supplementary Fig. 8. ^{13}C NMR spectrum of BTIC-ICBr in $CDCl_3$.

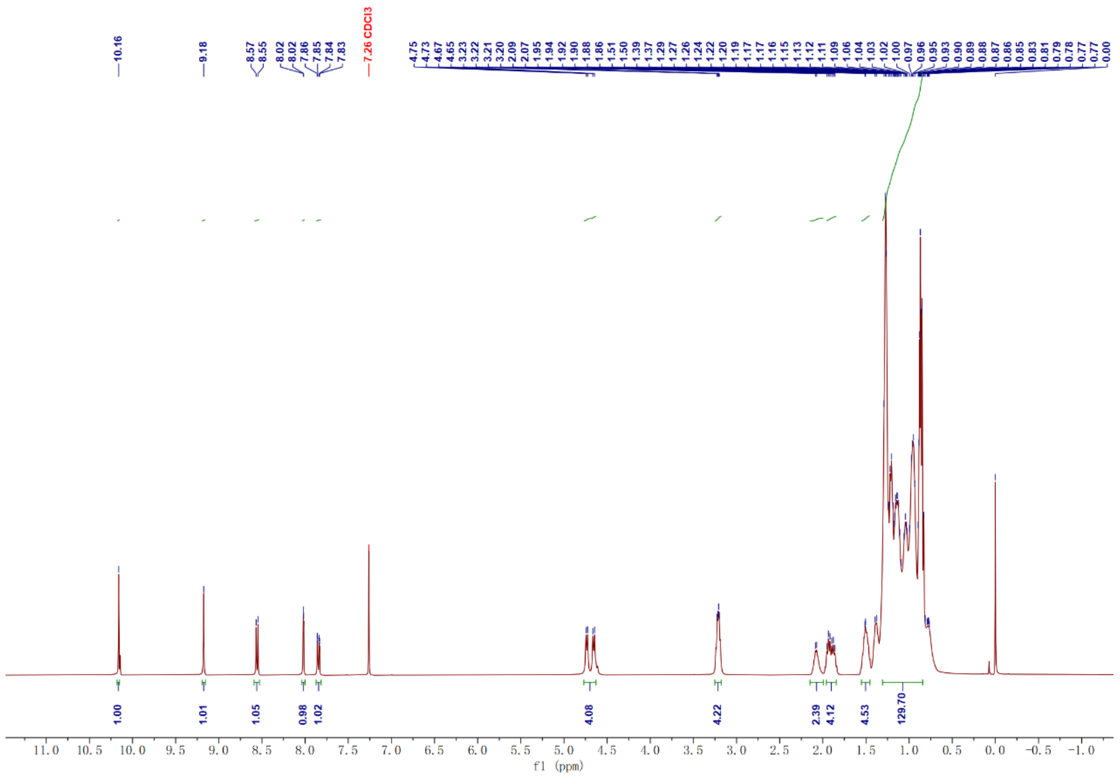

Supplementary Fig. 9. ^{1}H NMR spectrum of BTCHO-ICBr in $CDCl_3$.

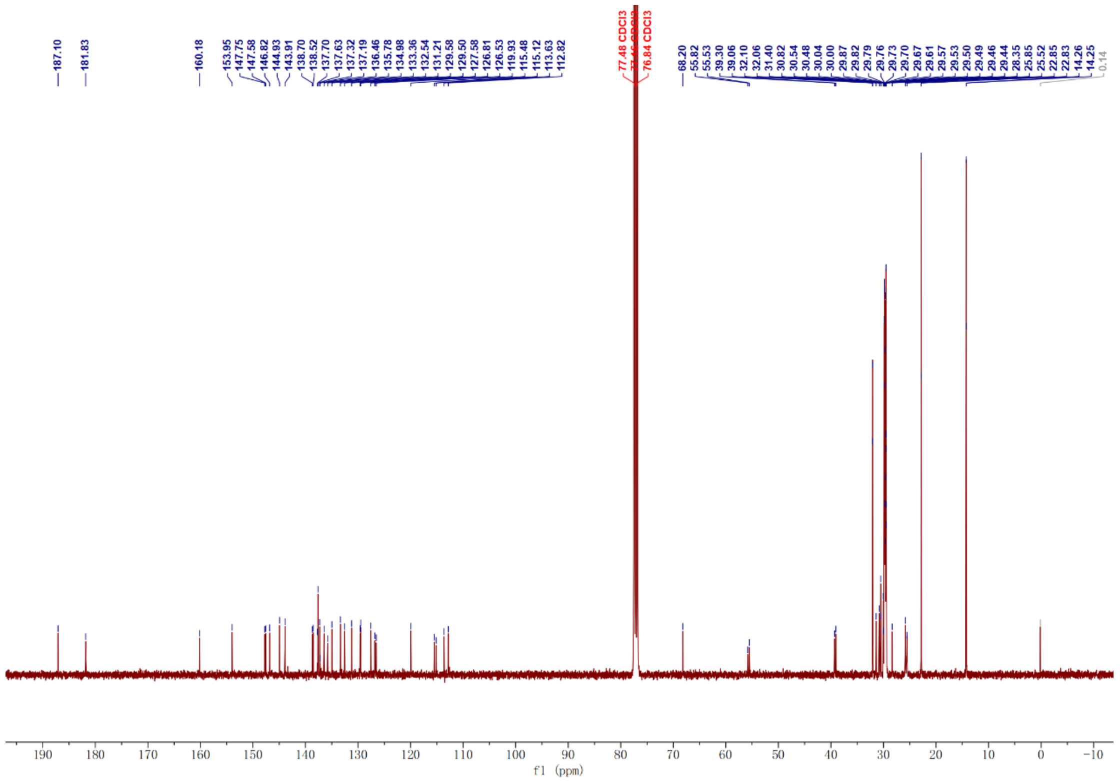

Supplementary Fig. 10. ^{13}C NMR spectrum of BTCHO-ICBr in $CDCl_3$.

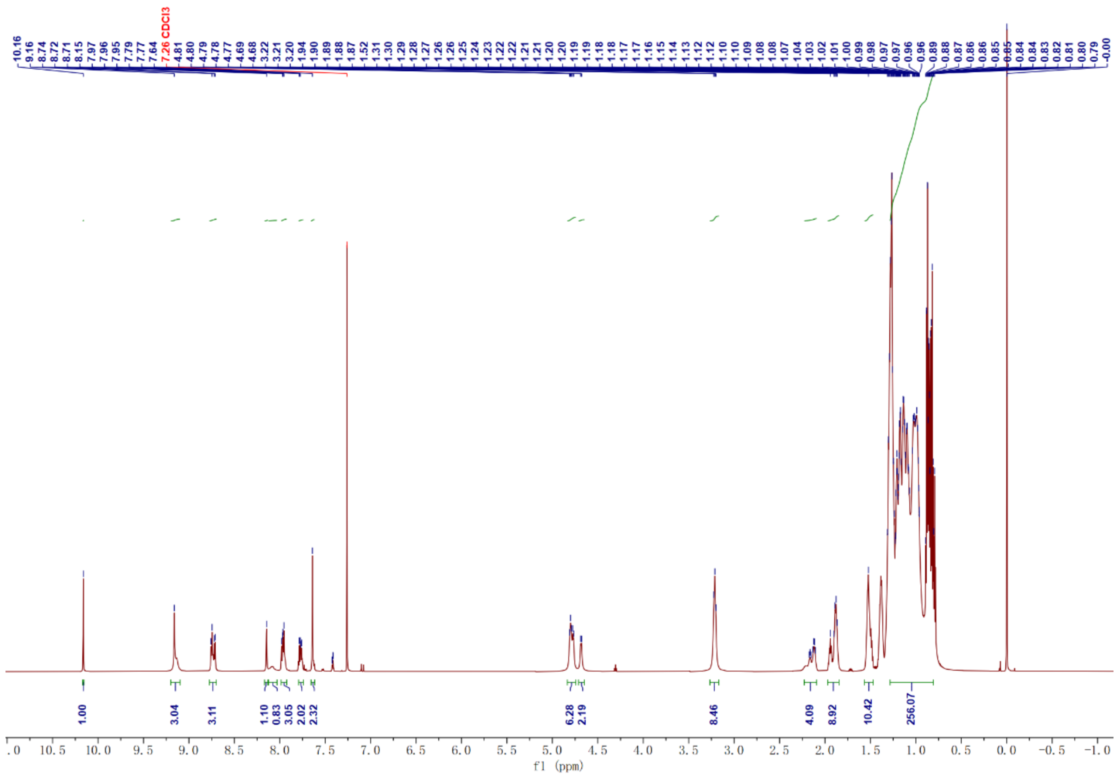

Supplementary Fig. 11. ^{1}H NMR spectrum of 2BTCHO-IC in $CDCl_3$.

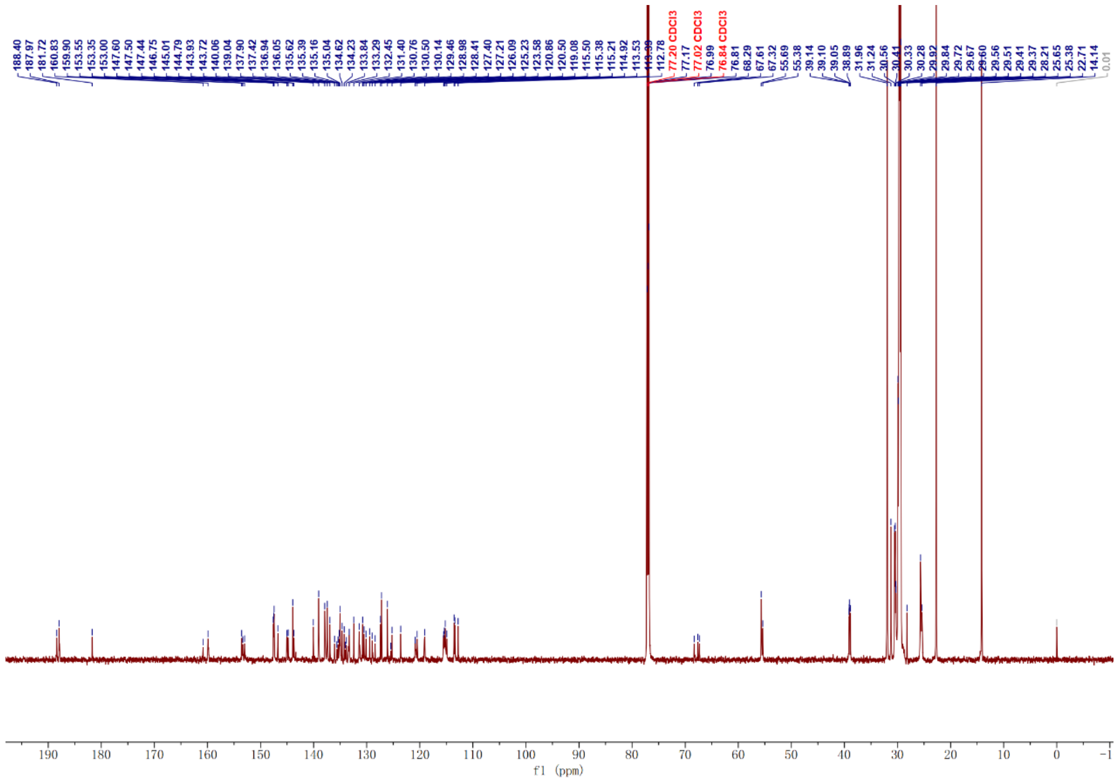

Supplementary Fig. 12. ^{13}C NMR spectrum of 2BTCHO-IC in $CDCl_3$.

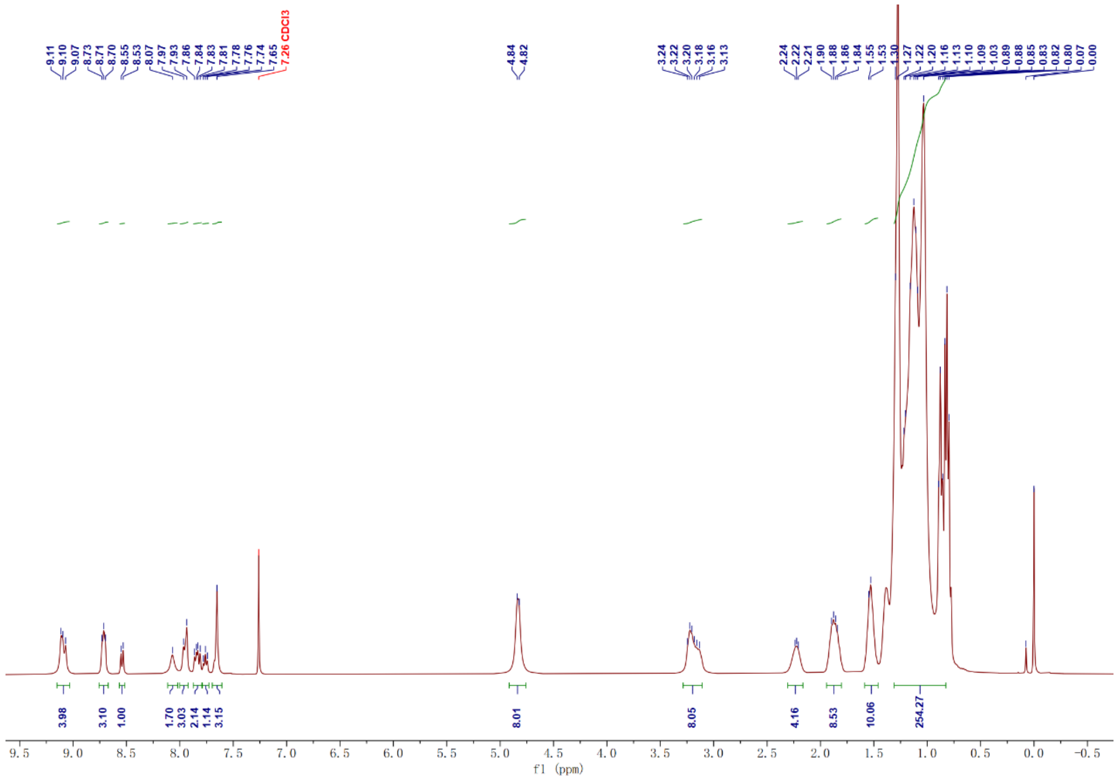

Supplementary Fig. 13. ^{1}H NMR spectrum of 2BTIC-ICBr in $CDCl_3$.

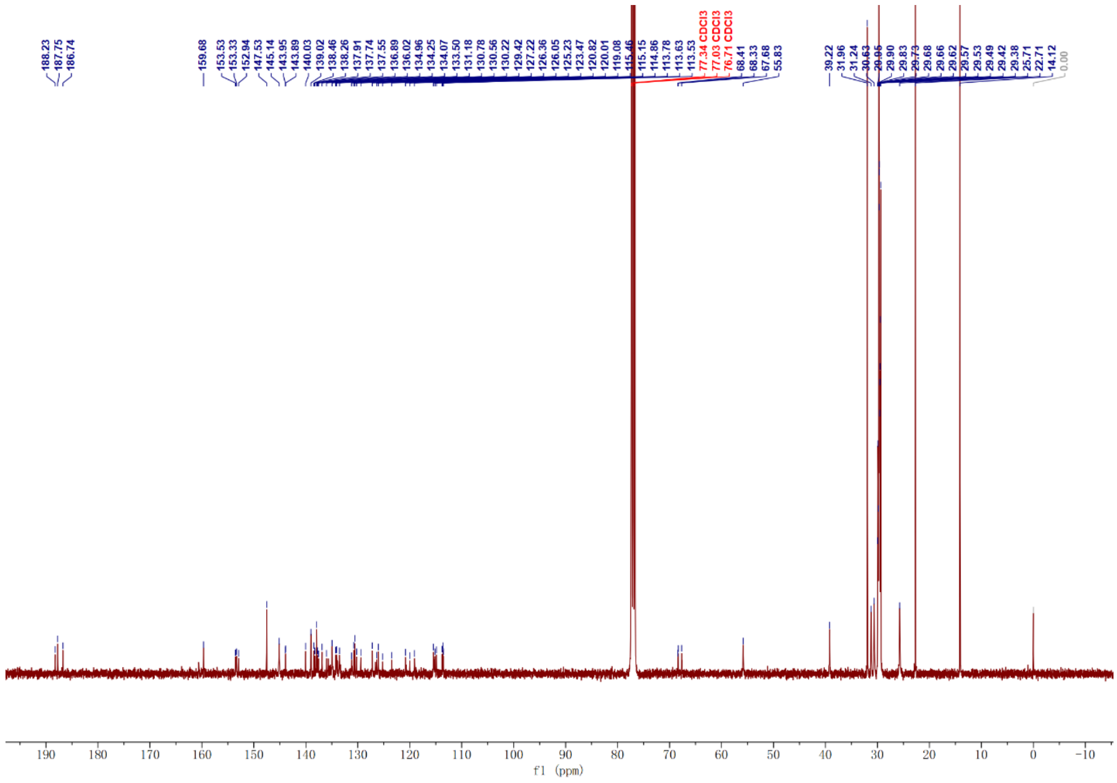

Supplementary Fig. 14. ^{13}C NMR spectrum of 2BTIC-ICBr in $CDCl_3$.

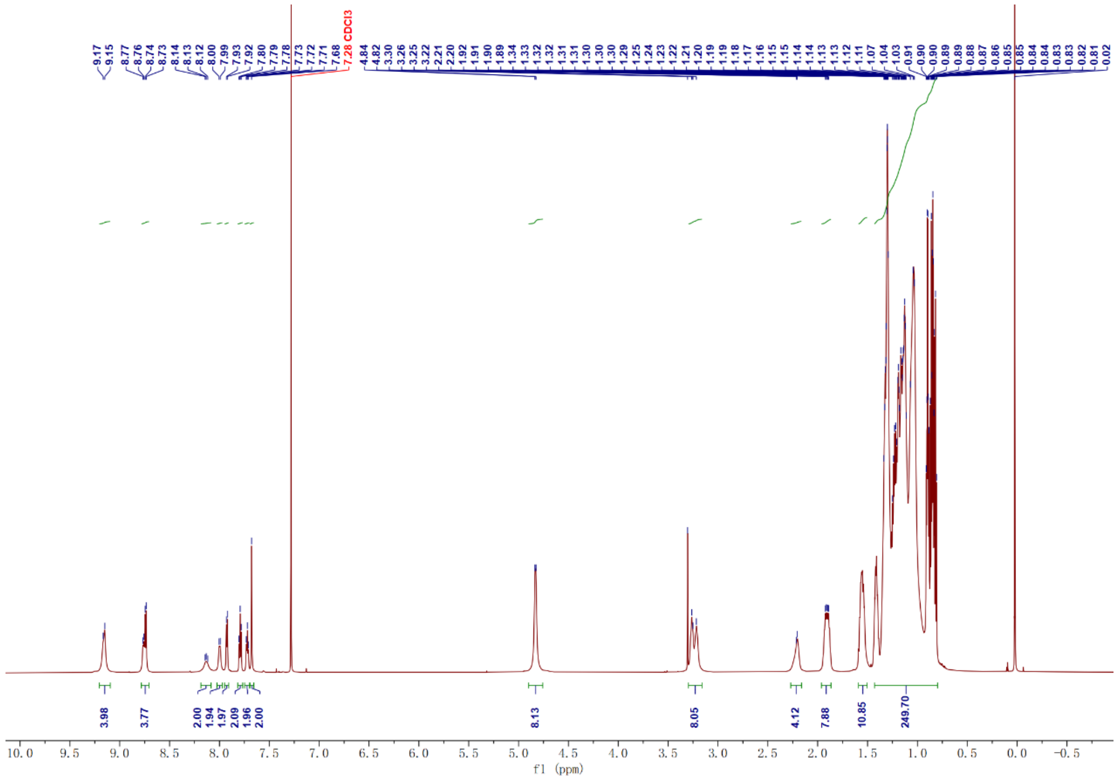

Supplementary Fig. 15. ^{1}H NMR spectrum of DY in $CDCl_3$.

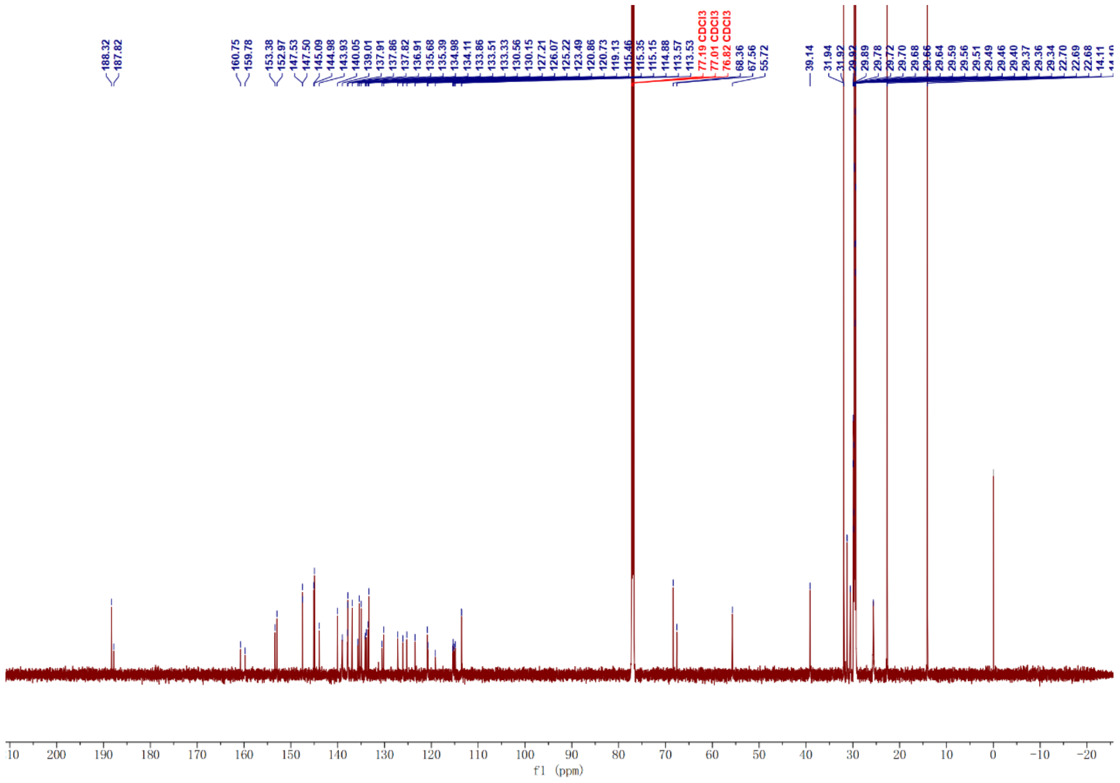

Supplementary Fig. 16. ^{13}C NMR spectrum of DY in $CDCl_3$.

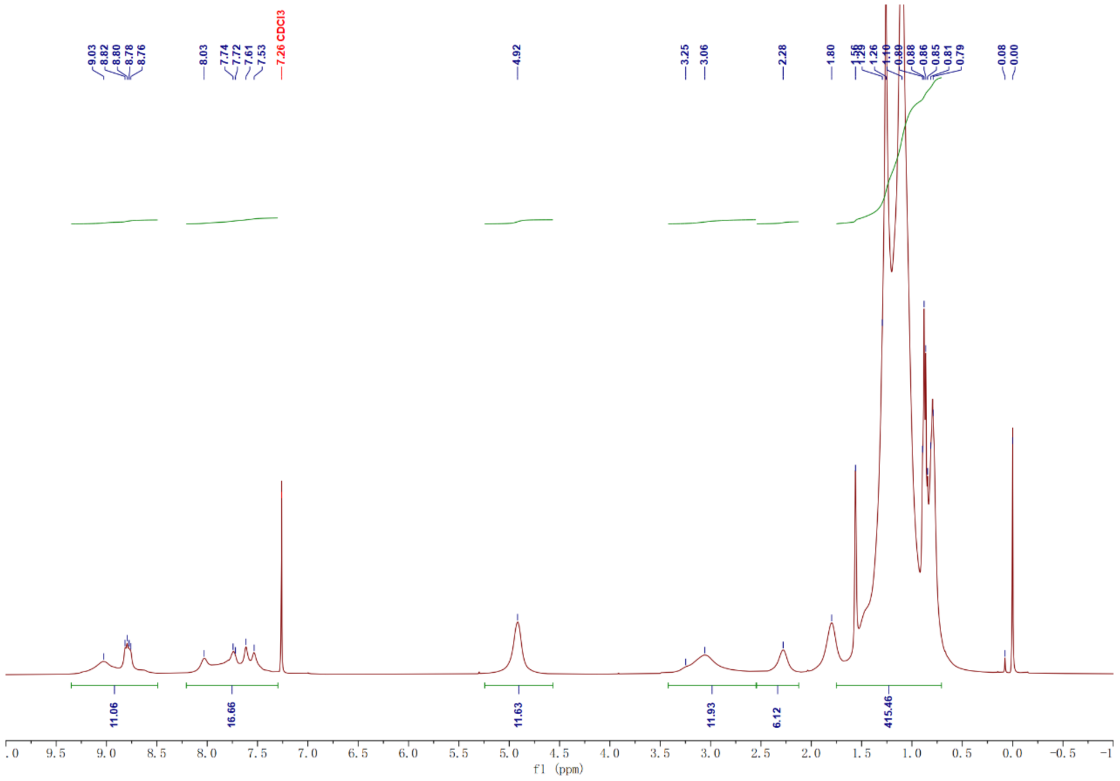

Supplementary Fig. 17. ^{1}H NMR spectrum of TY in $CDCl_3$.

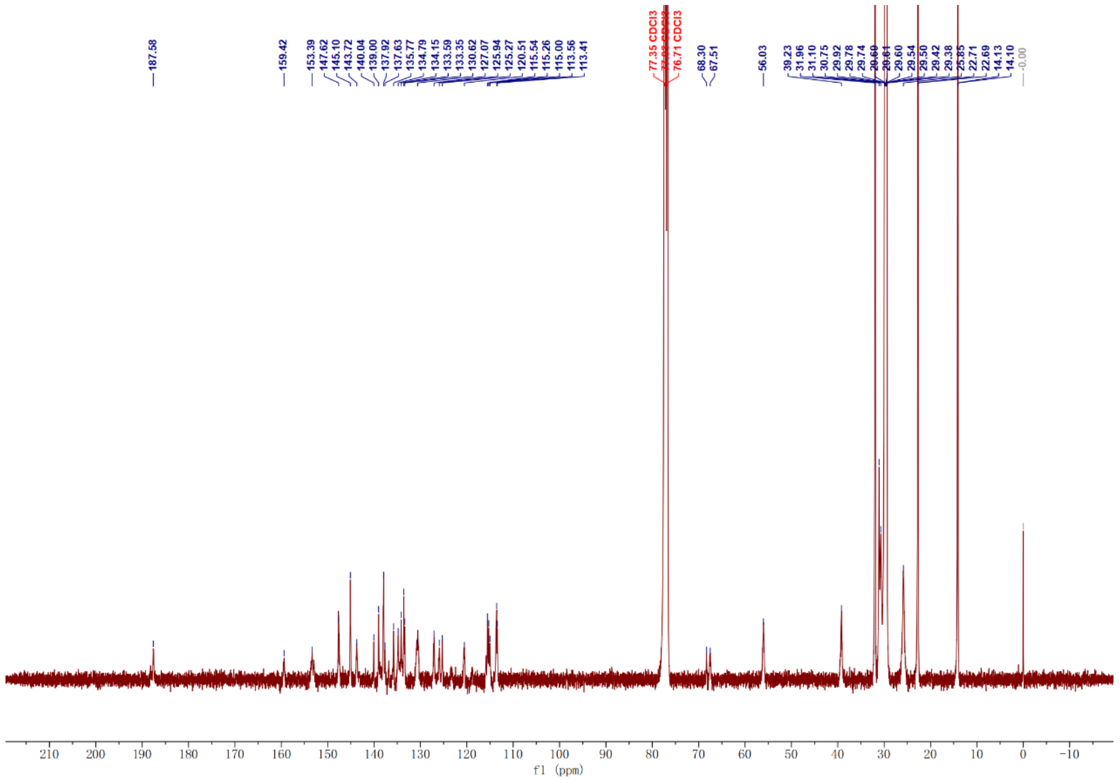

Supplementary Fig. 18. ^{13}C NMR spectrum of TY in $CDCl_3$.

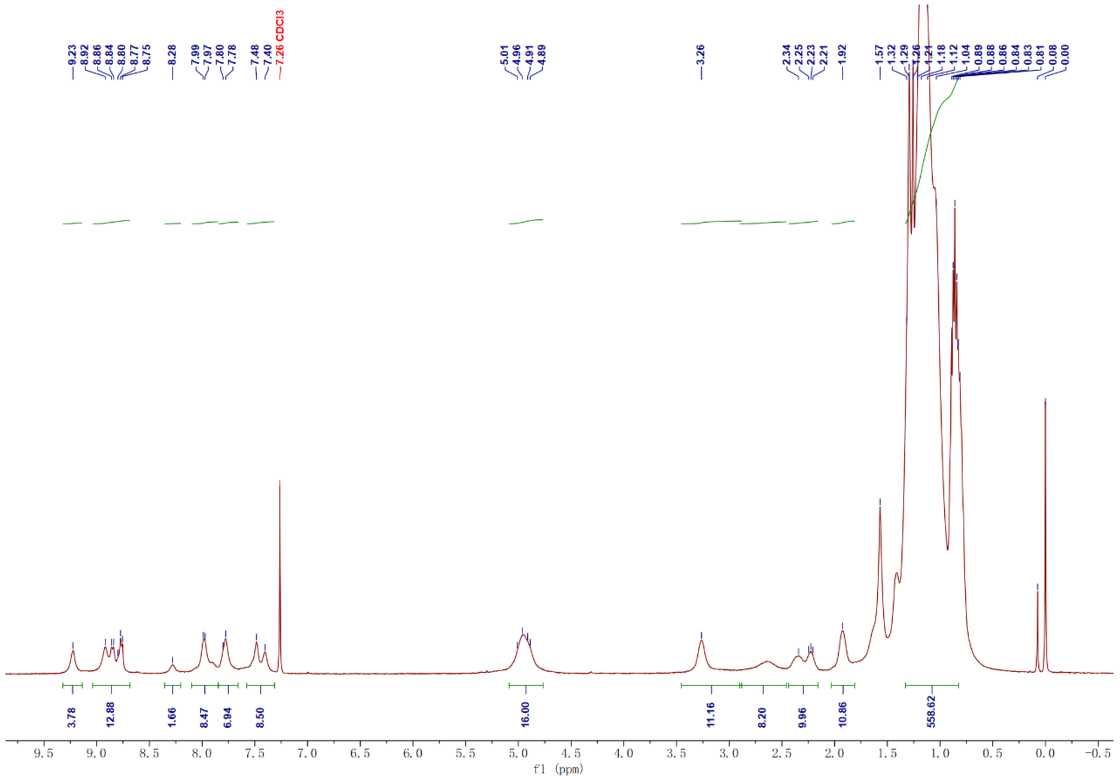

Supplementary Fig. 19. ^{1}H NMR spectrum of QY in $CDCl_3$.

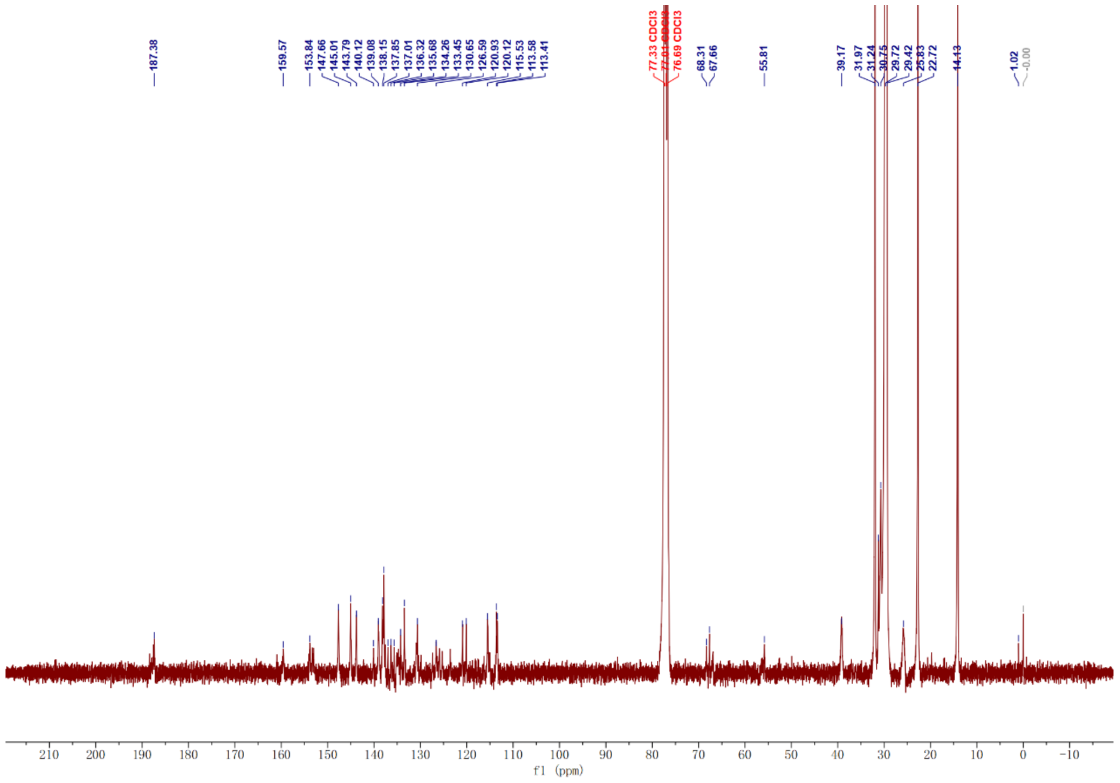

Supplementary Fig. 20. ^{13}C NMR spectrum of QY in $CDCl_3$.

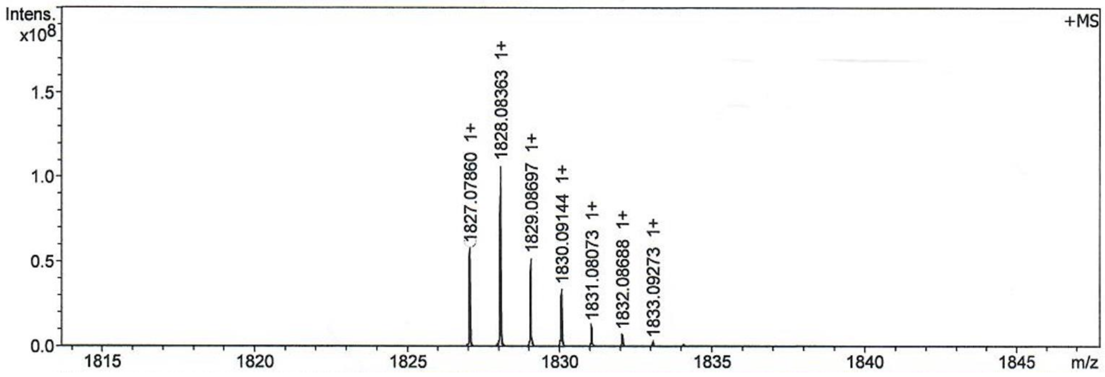

Supplementary Fig. 21. MALDI-TOF-MS Result of YDT.

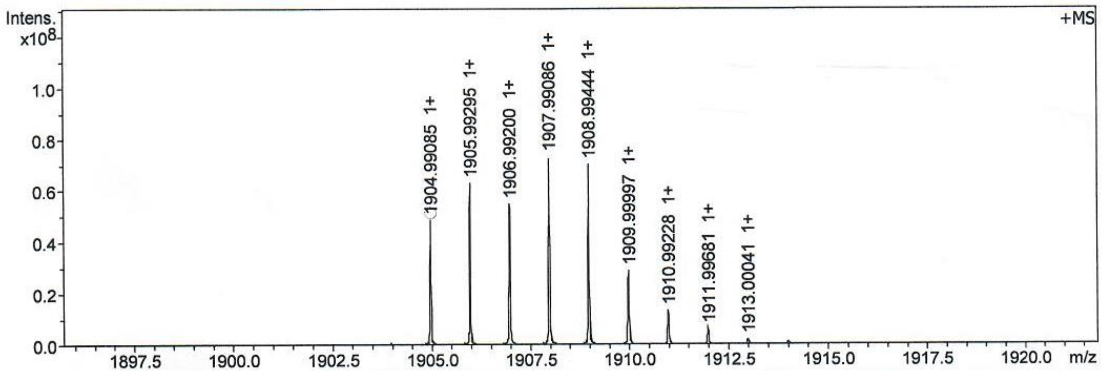

Supplementary Fig. 22. MALDI-TOF-MS Result of BTIC-ICBr.

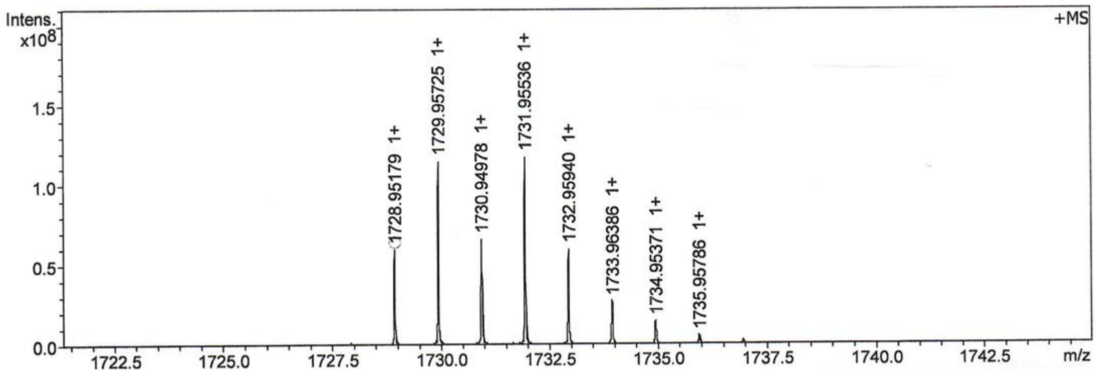

Supplementary Fig. 23. MALDI-TOF-MS Result of BTCHO-ICBr.

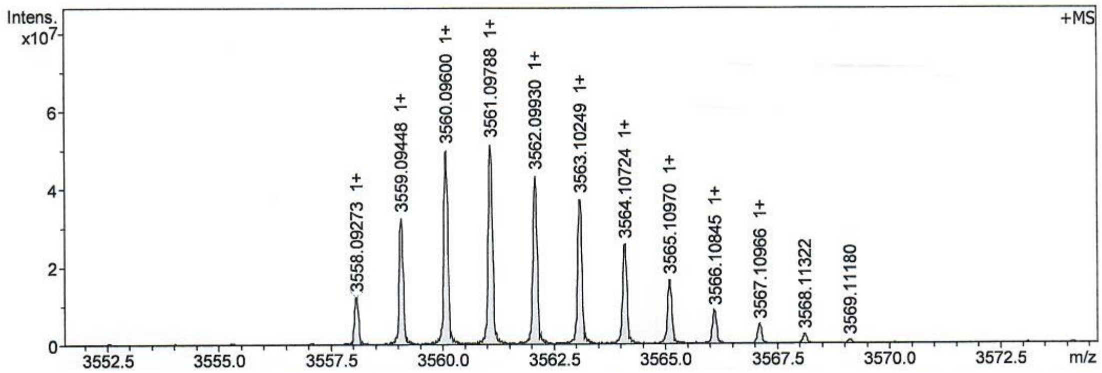

Supplementary Fig. 24. MALDI-TOF-MS Result of 2BTCHO-IC.

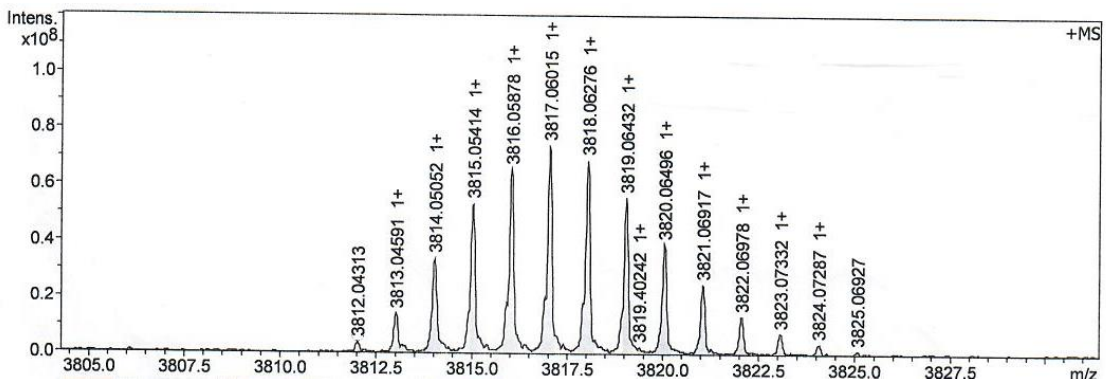

Supplementary Fig. 25. MALDI-TOF-MS Result of 2BTIC-ICBr.

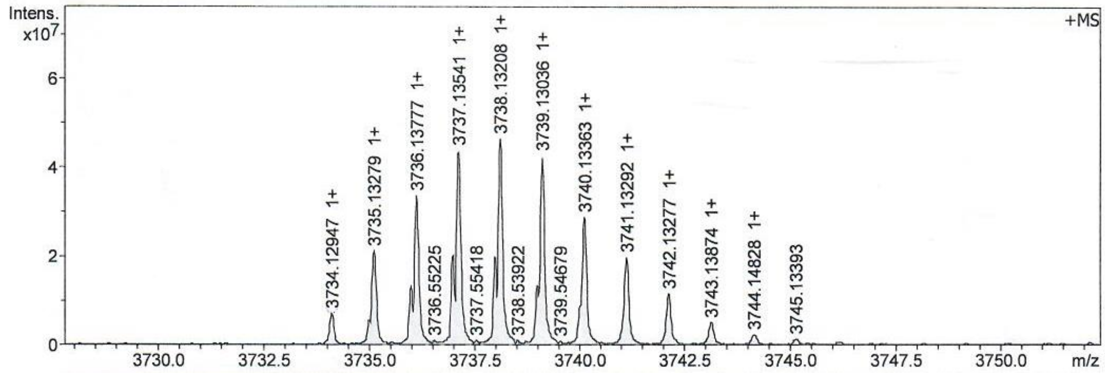

Supplementary Fig. 26. MALDI-TOF-MS Result of DY.

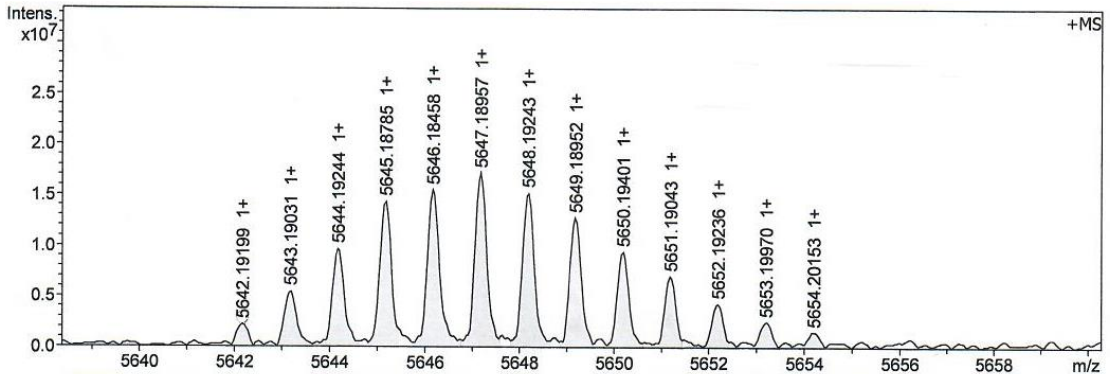

Supplementary Fig. 27. MALDI-TOF-MS Result of TY.

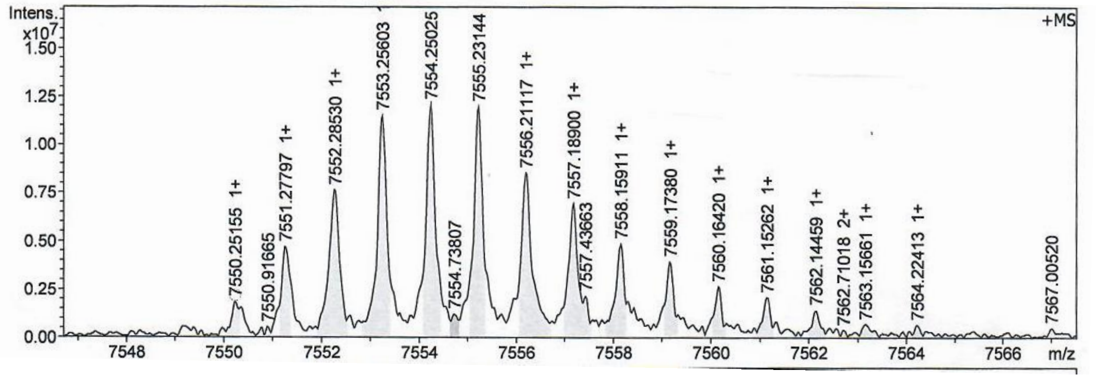

Supplementary Fig. 28. MALDI-TOF-MS Result of QY.

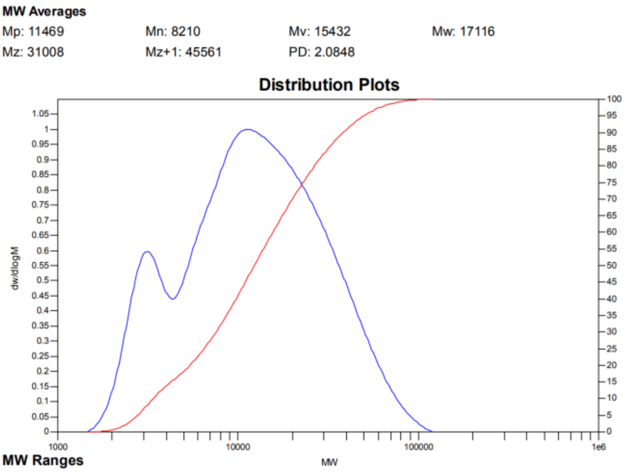

Supplementary Fig. 29. High-temperature GPC results of PY-IT.

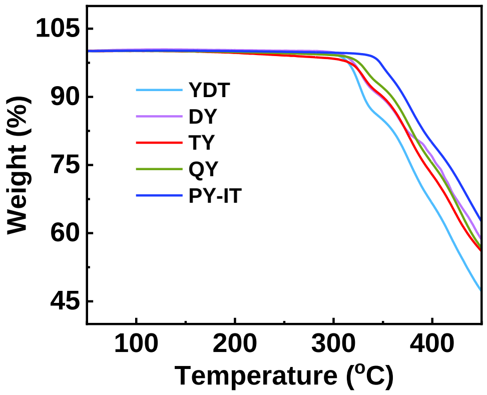

Supplementary Fig. 30. Thermogravimetric analysis (TGA) plots of YDT, DY, TY, QY and PY-IT.

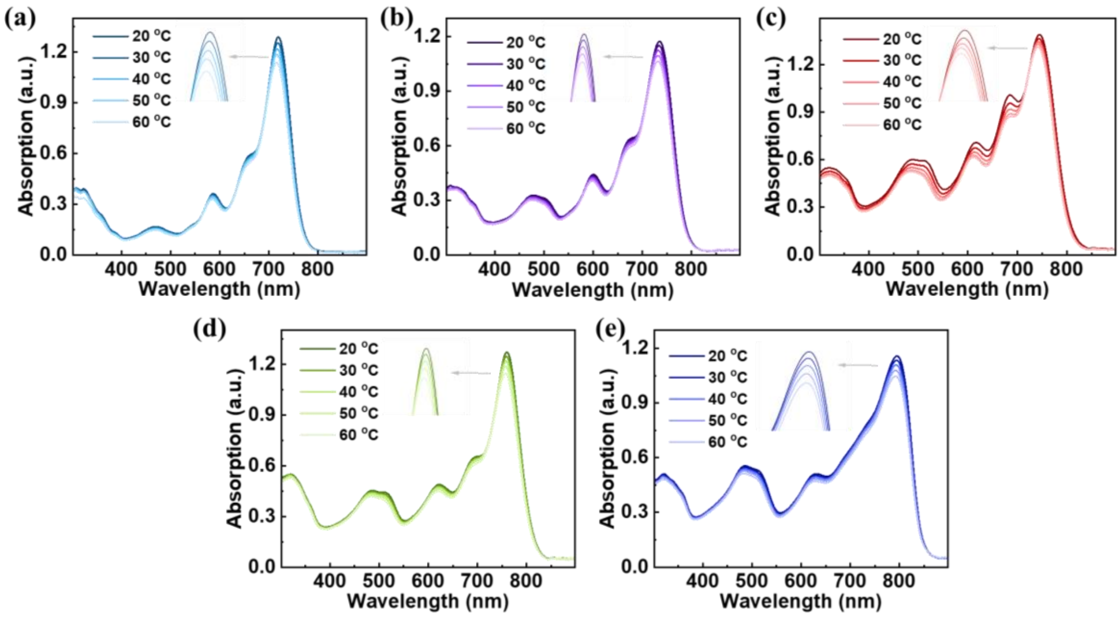

Supplementary Fig. 31. Variable-temperature UV-vis absorption spectra of (a) YDT, (b) DY, (c) TY, (d) QY and (e) PY-IT in chloroform.

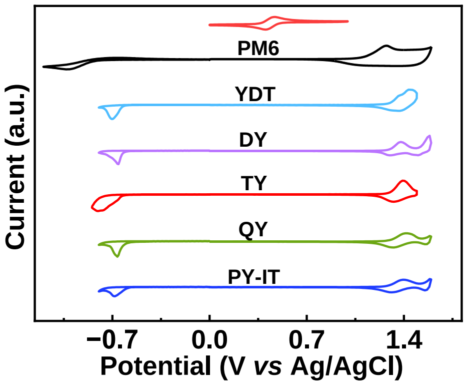

Supplementary Fig. 32. Cyclic voltammograms of PM6 and the corresponding acceptors.

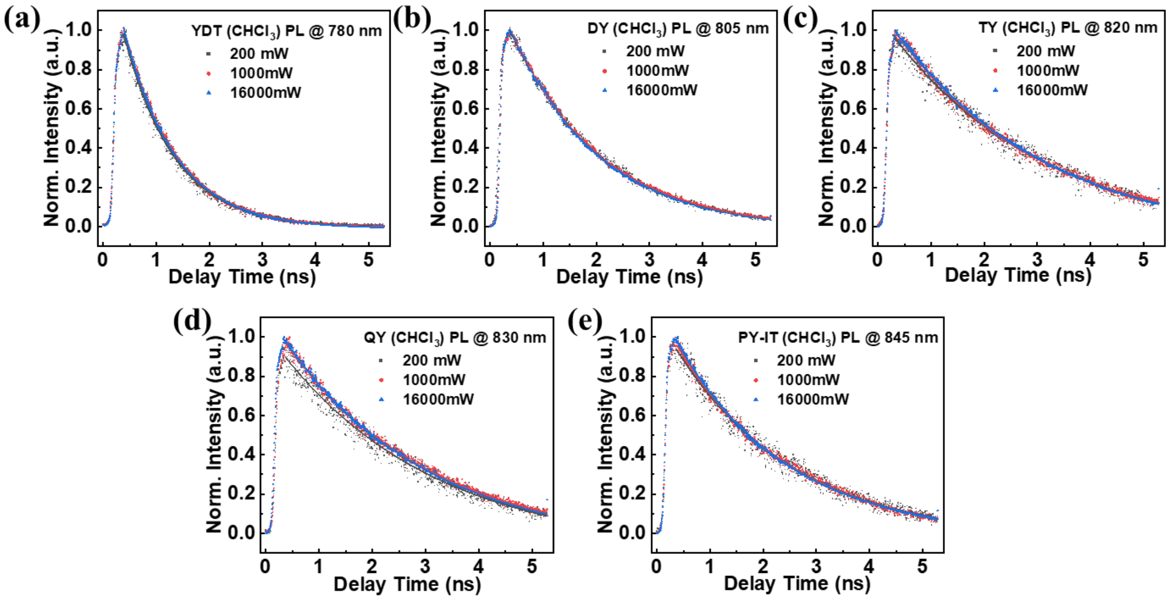

Supplementary Fig. 33. Time resolved photoluminescence spectra of (a) YDT, (b) DY, (c) TY, (d) QY and (e) PY-IT in diluted chloroform solution with various excitation intensities. The PL decays are fluence independent for all samples in diluted solution, which means annihilation does not occur. Lifetimes were well fitted using mono-exponential decay function.

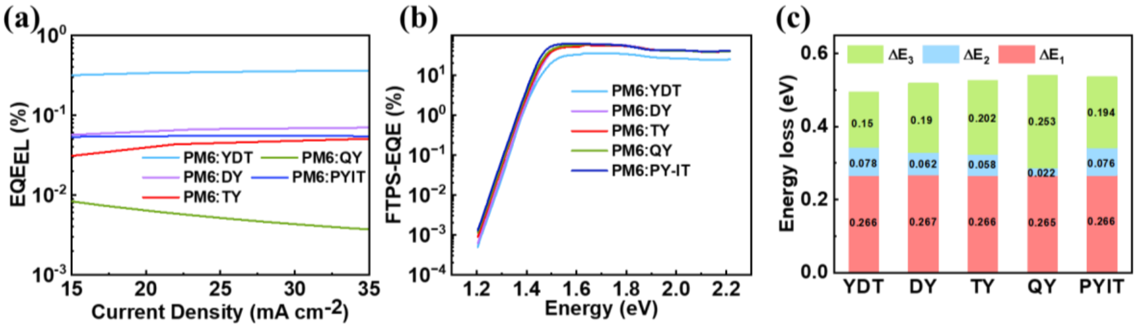

Supplementary Fig. 34. (a) The electroluminescence quantum efficiency at different injected currents, (b) the Fourier-transform photocurrent spectroscopy and (c) the energy loss for the optimized devices based on YDT, DY, TY, QY and PY-IT, respectively.

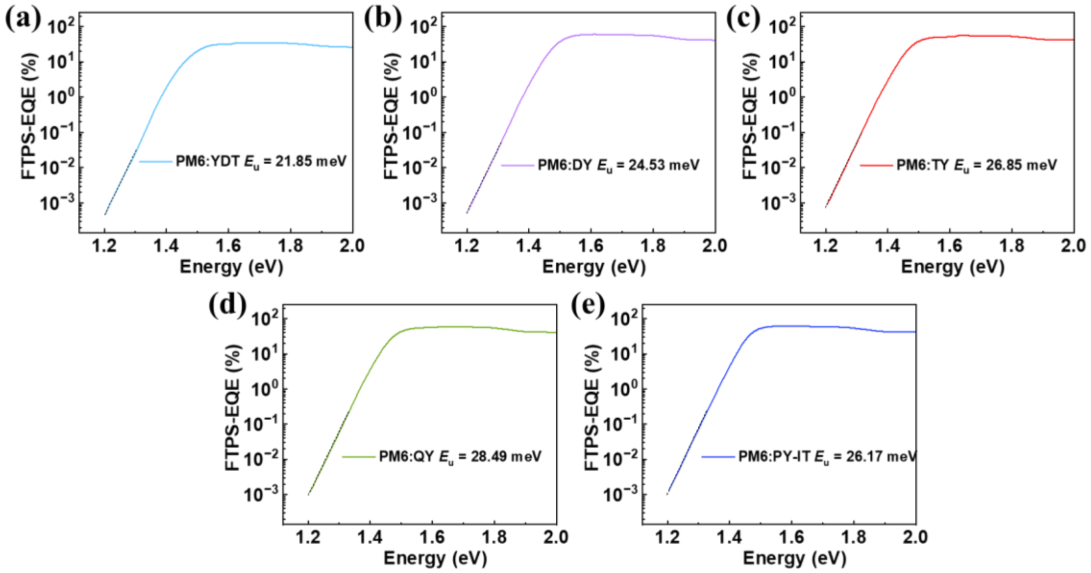

Supplementary Fig. 35. FTPS-EQE of the devices based on (a) PM6:YDT, (b) PM6:DY, (c) PM6:TY, (d) PM6:QY and (e) PM6:PY-IT at the absorption onset.

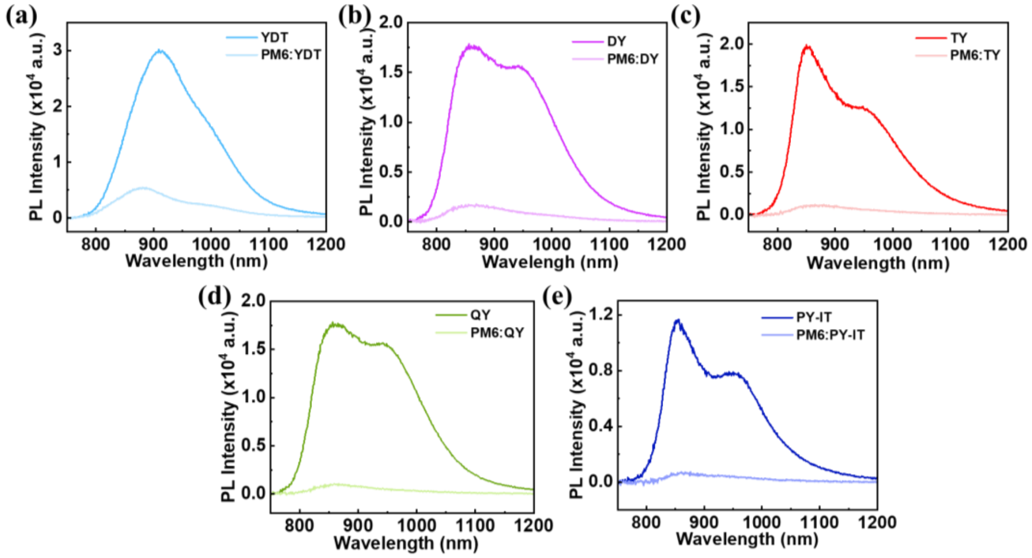

Supplementary Fig. 36. Fluorescence spectra of neat and blend films of (a) YDT, (b) DY, (c) TY, (d) QY and (e) PY-IT excited at 700 nm.

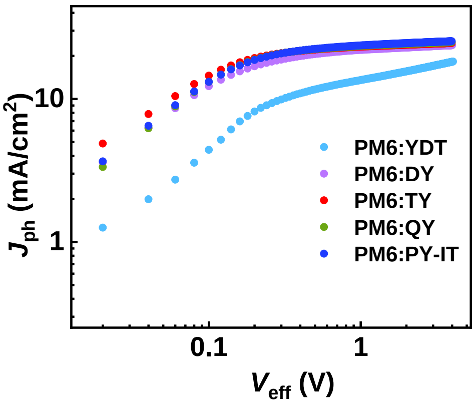

Supplementary Fig. 37. J_{ph} *vs* V_{eff} curves of the optimized OSCs based on PM6:acceptors.

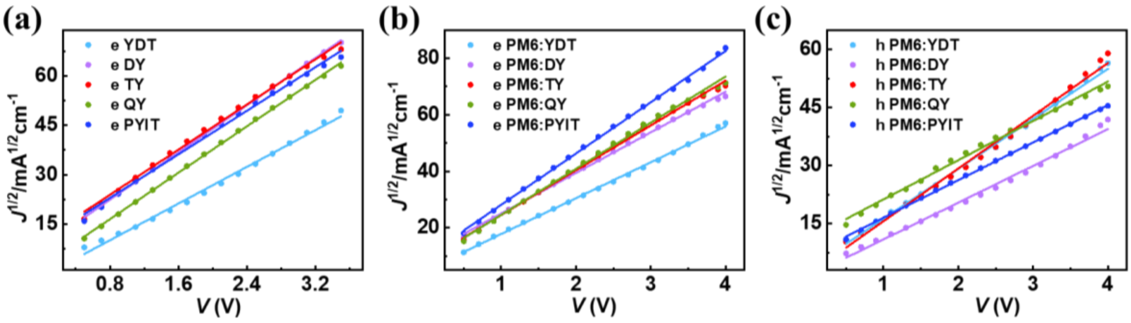

Supplementary Fig. 38. Measurement plots for (a) electron mobility of the acceptor neat films, (b) electron mobility of the blend active layers and (c) hole mobility of the blend active layers.

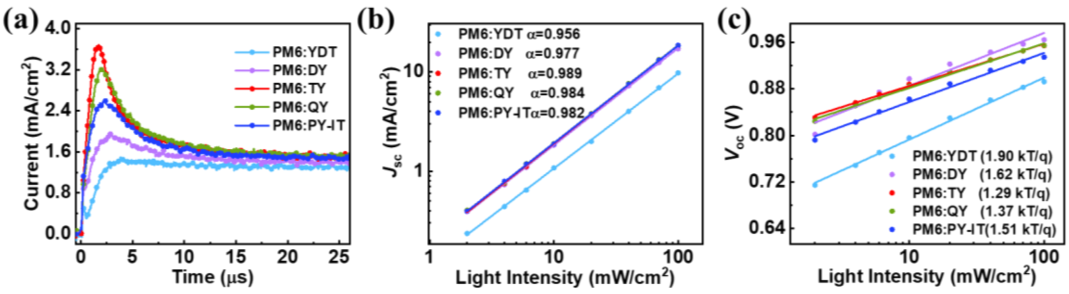

Supplementary Fig. 39. (a) Photo-CELIV curves of the optimized OSCs based on PM6:acceptors. The dependences of (b) J_{sc} and (c) V_{oc} on light intensity of the optimized devices.

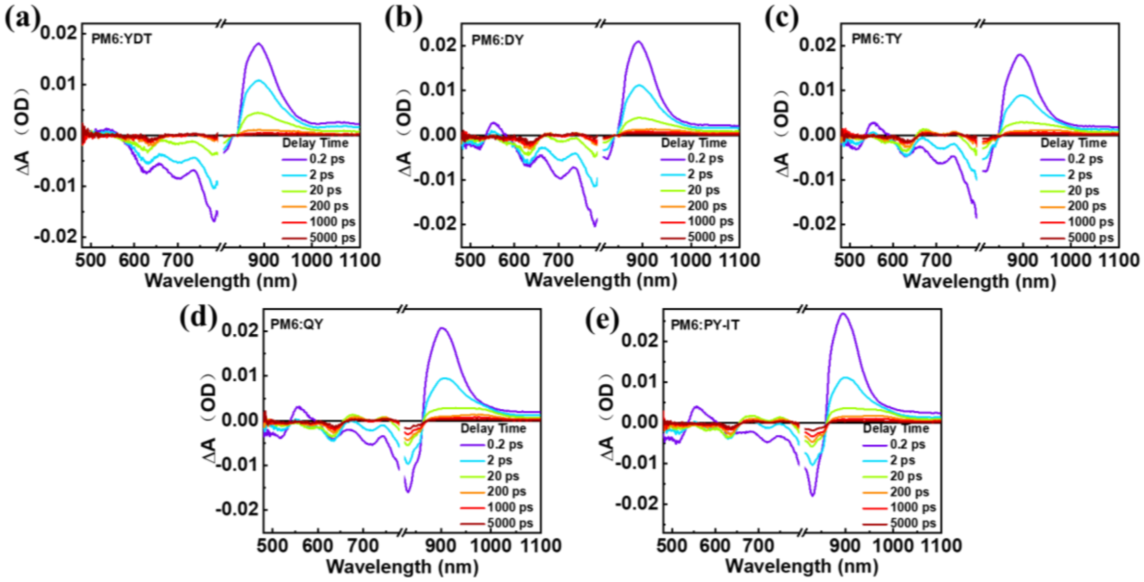

Supplementary Fig. 40. Femtosecond transient absorption spectra of (a) PM6:YDT blend film, (b) PM6:DY blend film, (c) PM6:TY blend film, (d) PM6:QY blend film and (e) PM6:PY-IT blend film at selected time delays.

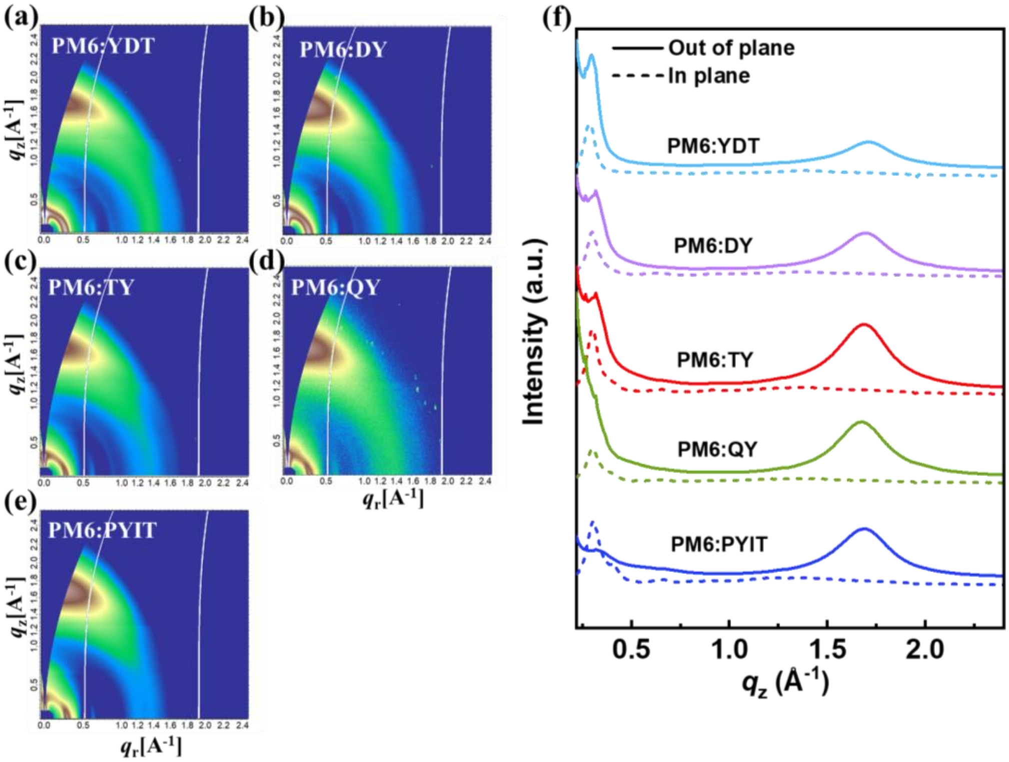

Supplementary Fig. 41. The 2D GIWAXS patterns of the blend films for (a) PM6:YDT, (b) PM6:DY, (c) PM6:TY, (d) PM6:QY and (e) PM6:PY-IT; (f) In-plane and out-of-plane line-cuts of the corresponding 2D GIWAXS patterns.

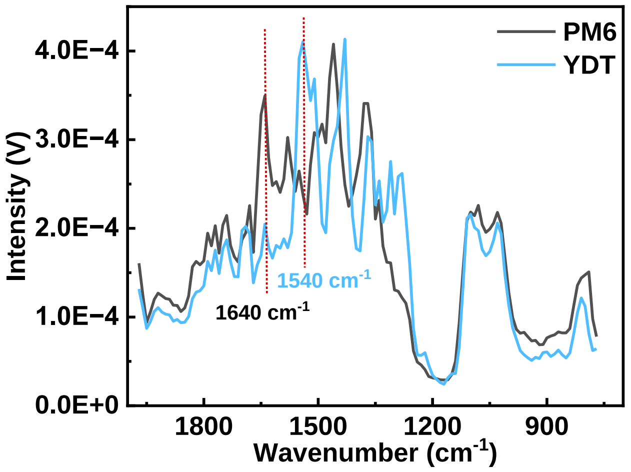

Supplementary Fig. 42. The PiFM infrared (IR) spectra of neat PM6 and YDT films.

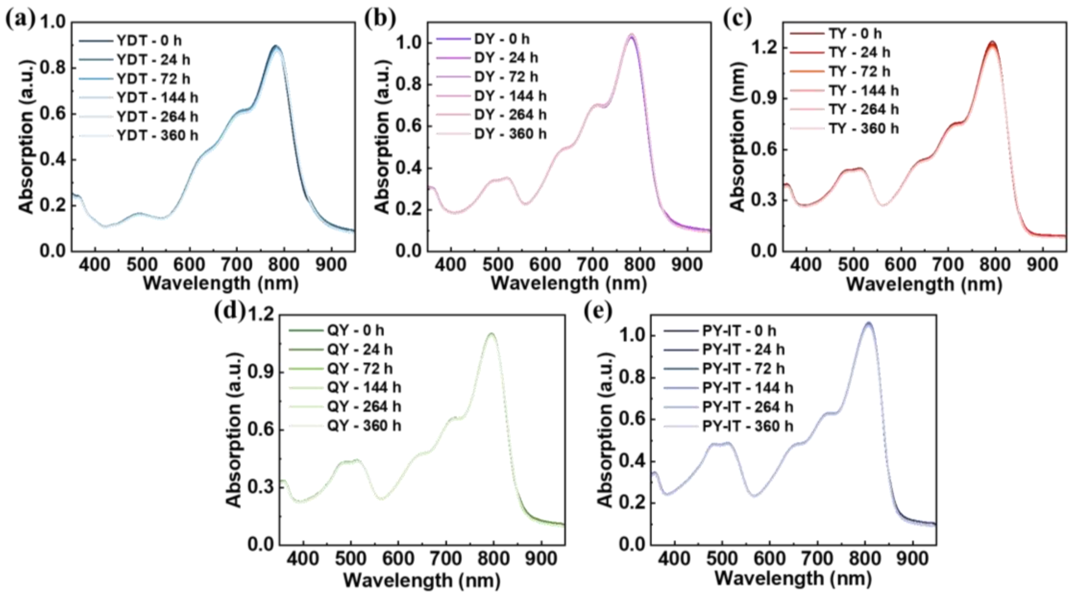

Supplementary Fig. 43. The UV-vis absorption spectra of (a) YDT, (b) DY, (c) TY, (d) QY and (e) PY-IT neat films before and after 360 h under one-sun-equivalent illumination in nitrogen filled glove box.

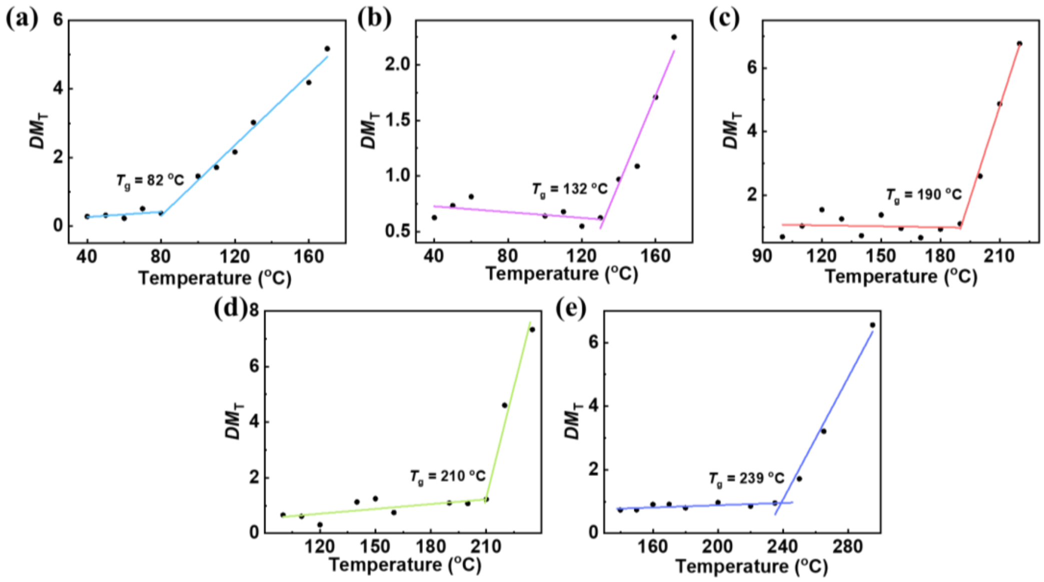

Supplementary Fig. 44. Plots of DM_T of (a) YDT, (b) DY, (c) TY, (d) QY and (e) PY-IT films as a function of annealing temperature.

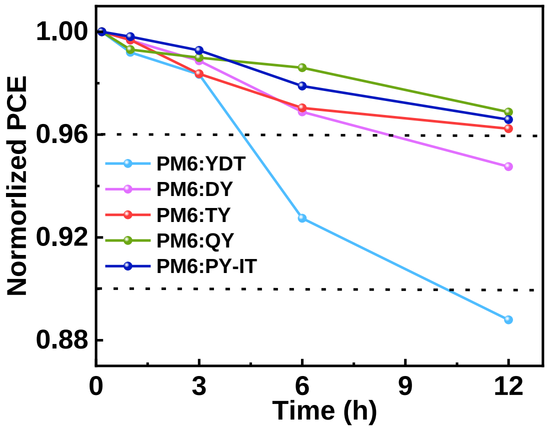

Supplementary Fig. 45. The thermal stability of photovoltaic performance of the OSCs based on PM6:YDT/DY/TY/QY/PY-IT active layers under the continuous thermal annealing at 90 °C.

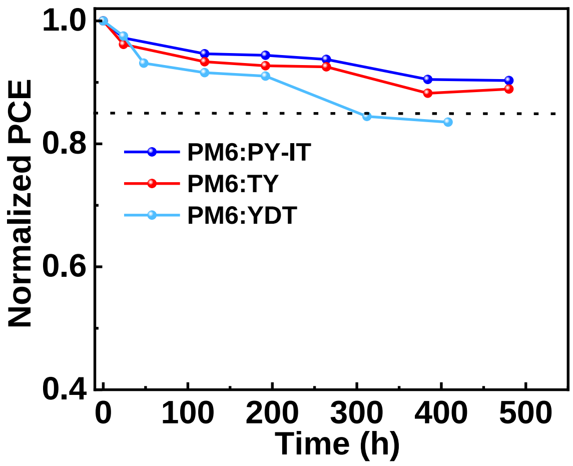

Supplementary Fig. 46. The PCE decay curves of PM6:YDT, PM6:TY and PM6:PY-IT cells under different aging time at the RT in nitrogen filled glove box.

Supplementary Tables

Supplementary Table 1. Energy loss of the devices based on PM6:YDT/DY/TY/QY/PY-IT.

Devices	$E_g^{PV\ c)}$ (eV)	EQE_{EL}	qV_{oc}^{SQ} (eV)	qV_{oc}^{rad} (eV)	ΔE_{loss} (eV)	ΔE_1 (eV)	ΔE_2 (eV)	ΔE_3 (eV)	V_{oc}^{Cal} (V)
PM6:YDT	1.482	3.08×10^{-3}	1.216	1.138	0.494	0.266	0.078	0.15	0.988
PM6:DY	1.481	6.37×10^{-4}	1.214	1.152	0.519	0.267	0.062	0.19	0.962
PM6:TY	1.475	4.10×10^{-4}	1.209	1.151	0.526	0.266	0.058	0.202	0.949
PM6:QY	1.476	5.78×10^{-5}	1.211	1.189	0.540	0.265	0.022	0.253	0.936
PM6:PY-IT	1.465	5.47×10^{-4}	1.200	1.123	0.536	0.266	0.076	0.194	0.929

Supplementary Table 2. Detailed data of the hole mobility (μ_h) and electron mobility (μ_e).

Active layer	μ_h ($cm^2\ Vs^{-1}$)	μ_e ($cm^2\ Vs^{-1}$)	μ_h/μ_e
YDT	-	8.6×10^{-4}	-
DY	-	13.9×10^{-4}	-
TY	-	17.1×10^{-4}	-
QY	-	15.8×10^{-4}	-

PY-IT	-	14.4×10^{-4}	-
PM6:YDT	19.3×10^{-4}	9.3×10^{-4}	2.07
PM6:DY	15.97×10^{-4}	11.92×10^{-4}	1.34
PM6:TY	21.6×10^{-4}	17.56×10^{-4}	1.23
PM6:QY	17.34×10^{-4}	13.54×10^{-4}	1.28
PM6:PY-IT	18.6×10^{-4}	12.65×10^{-4}	1.47

Supplementary Table 3. Molecular packing parameters for neat and blend films derived from GIWAXS fitting.

Films	Directions	Diffraction	Location (Å^{-1})	d-spacing (Å)	FWHM (Å^{-1})	CCL[a] (Å)
PM6	In-plane	(100)	0.335	18.746	0.14	41.72
	Out-of-plane	(010)	1.704	3.685	0.35	16.69
YDT	In-plane	(100)	0.266	23.609	0.07	83.43
	Out-of-plane	(010)	1.686	3.725	0.36	16.22
DY	In-plane	(100)	0.306	20.523	0.23	25.39
	Out-of-plane	(010)	1.653	3.799	0.28	20.86
TY	In-plane	(100)	0.301	20.864	0.16	36.50
	Out-of-plane	(010)	1.648	3.811	0.28	20.86
QY	In-plane	(100)	0.371	16.927	0.18	32.45
	Out-of-plane	(010)	1.639	3.832	0.28	20.86
PY-IT	In-plane	(100)	0.387	16.227	0.19	30.74
	Out-of-plane	(010)	1.653	3.799	0.33	17.70
PM6:YDT	In-plane	(100)	0.286	21.958	0.07	83.43
	Out-of-plane	(010)	1.71	3.672	0.30	19.47
PM6:DY	In-plane	(100)	0.301	20.864	0.06	97.34
	Out-of-plane	(010)	1.69	3.716	0.32	18.25
PM6:TY	In-plane	(100)	0.301	20.864	0.06	97.34
	Out-of-plane	(010)	1.68	3.738	0.33	17.70

PM6:QY	In-plane	(100)	0.302	20.795	0.06	97.34
	Out-of-plane	(010)	1.67	3.760	0.32	18.25
PM6:PY-IT	In-plane	(100)	0.306	20.523	0.09	64.89
	Out-of-plane	(010)	1.68	3.738	0.33	17.70
