# Supplementary material for: Precise synthesis and photovoltaic properties of giant molecule acceptors
Source: Nat Commun. 2023 Dec 2;14:7996. doi: 10.1038/s41467-023-43846-3 (PMC10693637; doi:10.1038/s41467-023-43846-3)
Supplement: Supplementary file 3 — Reporting Summary [file 41467_2023_43846_MOESM3_ESM.pdf]

Corresponding author(s): Xiaojun Li, Harald Ade, Yongfang Li

Solar Cells Reporting Summary

Nature Research wishes to improve the reproducibility of the work that we publish. This form is intended for publication with all accepted papers reporting the characterization of photovoltaic devices and provides structure for consistency and transparency in reporting. Some list items might not apply to an individual manuscript, but all fields must be completed for clarity.

For further information on Nature Research policies, including our data availability policy, see Authors & Referees.

Experimental design

Please check: are the following details reported in the manuscript?

1. Dimensions

Item	Yes/No	Response
Area of the tested solar cells	☒ Yes ☐ No	Area of the tested solar cells is 0.06 cm2 defined by optical microscope (Olympus BX51). The description texts can be found in the Method Section.
Method used to determine the device area	☒ Yes ☐ No	Device area is determined by a mask with 0.0416 cm2

2. Current-voltage characterization

Item	Yes/No	Response
Current density-voltage (J-V) plots in both forward and backward direction	☐ Yes ☒ No	Generally, organic photovoltaic devices do not have forward and backward problems. And we only scan the device in forward direction.
Voltage scan conditions<br>*For instance: scan direction, speed, dwell times*	☒ Yes ☐ No	The voltage was scanned from -1.5 V to 2 V. The voltage step and delay time were 10 mV and 1 ms, respectively.
Test environment<br>*For instance: characterization temperature, in air or in glove box*	☒ Yes ☐ No	Devices were characterized at room temperature in In N2-filled glove box
Protocol for preconditioning of the device before its characterization	☐ Yes ☒ No	No preconditioning protocol.
Stability of the J-V characteristic<br>*Verified with time evolution of the maximum power point or with the photocurrent at maximum power point; see ref. 7 for details.*	☐ Yes ☒ No	Organic photovoltaic devices show no decay or instability during the test of J-V characteristics. The MPP output is consistent with the J-V curve.

3. Hysteresis or any other unusual behaviour

Item	Yes/No	Response
Description of the unusual behaviour observed during the characterization	☐ Yes ☒ No	No hysteresis was observed in our device.
Related experimental data	☐ Yes ☒ No	We didn't find the unusual behaviour.

4. Efficiency

Item	Yes/No	Response
External quantum efficiency (EQE) or incident photons to current efficiency (IPCE)	☒ Yes ☐ No	The data is included in the manuscript. We show the EQE measurement details in the Methods section.
A comparison between the integrated response under the standard reference spectrum and the response measure under the simulator	☒ Yes ☐ No	The difference between the integrated current from EQE and the short-circuit current from J-V curve measured under AM 1.5G solar simulator is within 5% difference which is within the accuracy confidence of the measurements.
For tandem solar cells, the bias illumination and bias voltage used for each subcell	☐ Yes ☒ No	We did not make the tandem solar cells in this work.

5. Calibration

Item	Yes/No	Response
Light source and reference cell or sensor used for the characterization	☒ Yes ☐ No	Relative information is provided in method section.
Confirmation that the reference cell was calibrated and certified	☒ Yes ☐ No	Relative information is provided in method section.

Calculation of spectral mismatch between the reference cell and the devices under test

- [x] Yes
- [ ] No

Relative information is provided in method section.

6. Mask/aperture

Size of the mask/aperture used during testing

- [x] Yes
- [ ] No

Device area is determined by a mask with area of 0.0416 cm2.Relative information is provided in method section.

Variation of the measured short-circuit current density with the mask/aperture area

- [x] Yes
- [ ] No

The variation is within 0.3%

7. Performance certification

Identity of the independent certification laboratory that confirmed the photovoltaic performance

- [ ] Yes
- [x] No

The photovoltaic performance of our devices was not confirmed from independent certification laboratories.

A copy of any certificate(s)
Provide in Supplementary Information

- [ ] Yes
- [x] No

The photovoltaic performance of our devices was not confirmed from independent certification laboratories.

8. Statistics

Number of solar cells tested

- [x] Yes
- [ ] No

The average PCE of the PSCs is obtained from 10 independent devices

Statistical analysis of the device performance

- [x] Yes
- [ ] No

We have given statistical data of device performance.

9. Long-term stability analysis

Type of analysis, bias conditions and environmental conditions
For instance: illumination type, temperature, atmosphere humidity, encapsulation method, preconditioning temperature

- [ ] Yes
- [x] No

We didn't make the long-term stability measurement for the devices.
